# Evidence for Human-to-Human Transmission of Hantavirus: A Systematic Review^[Author-notes jiab461-FM1]^

**DOI:** 10.1093/infdis/jiab461

**Published:** 2021-09-13

**Authors:** Joao Toledo, Michelle M Haby, Ludovic Reveiz, Leopoldo Sosa Leon, Rodrigo Angerami, Sylvain Aldighieri

**Affiliations:** Department of Health Emergencies, Pan American Health Organization, Washington, District of Columbia, USA; Departamento de Ciencias Químico Biológicas, Universidad de Sonora, Hermosillo, Sonora, Mexico; Department of Evidence and Intelligence for Action in Health, Pan American Health Organization, Washington, District of Columbia, USA; Independent consultant, Hermosillo, Sonora, Mexico; Hospital Epidemiology Section, Hospital of Clinics, State University of Campinas, Campinas, São Paulo, Brazil; Hospital Epidemiology Section, Hospital of Clinics, State University of Campinas, Campinas, São Paulo, Brazil

**Keywords:** hantavirus, hantavirus pulmonary syndrome, infectious disease transmission, hemorrhagic fever with renal syndrome, healthcare-associated infections, outbreak, systematic review

## Abstract

**Background:**

Hantavirus is known to be transmitted from rodents to humans. However, some reports from Argentina and Chile have claimed that the hantavirus strain Andes virus (ANDV) can cause human-to-human transmission of the disease. The aim of this systematic review was to assess the evidence for human-to-human transmission of hantavirus.

**Methods:**

We searched PubMed (inception to 28 February 2021), Cochrane Central, Embase, LILACS and SciELO (inception to 3 July 2020), and other sources. We included studies that assessed whether interpersonal contact with a person with laboratory-confirmed hantavirus infection led to human-to-human transmission. Two reviewers conducted screening, selection, data extraction, and risk of bias assessment.

**Results:**

Twenty-two studies met the inclusion criteria. Meta-analysis was not possible due to heterogeneity. With the exception of 1 prospective cohort study of ANDV in Chile with serious risk of bias, evidence from comparative studies (strongest level of evidence available) does not support human-to-human transmission of hantavirus infection. Noncomparative studies with a critical risk of bias suggest that human-to-human transmission of ANDV may be possible.

**Conclusions:**

The balance of the evidence does not support the claim of human-to-human transmission of ANDV. Well-designed cohort and case-control studies that control for co-exposure to rodents are needed to inform public health recommendations.

## Teaser

The balance of the evidence does not support the claim that human-to-human transmission of hantavirus infection occurs. Well-designed cohort and case-control studies that control for co-exposure to rodents are needed to inform public health recommendations.

The 1993 hantavirus outbreak in the southwest of the United States [1] led to 27 cases identified as hantavirus pulmonary syndrome (HPS) and sparked considerable research in the years that followed. To date, much is known and generally accepted about hantaviruses. While more hantavirus research will no doubt continue to emerge, thereby deepening our understanding of the subject matter as a whole, there remains a somewhat controversial and contestable issue, namely the claim that a particular hantavirus strain can cause human-to-human transmission of HPS.

Hantaviruses (family Hantaviridae, genus *Orthohantavirus*) cause 2 zoonotic diseases in humans, which are clinically manifested in 2 distinct forms: (1) hemorrhagic fever with renal syndrome (HFRS), which occurs in Europe and Asia; and (2) HPS in the Americas. Typically, HFRS causes a mild-to-moderate infection presenting fever, headaches, and gastrointestinal symptoms, with a progression to hypotension and acute renal failure, but with a low fatality rate (1%–15%) [2, 3]. HPS, by contrast, shows a noncardiogenic pulmonary edema and subsequent respiratory compromise, without the renal component of HFRS and a fatality rate of up to 60% [4, 5].

Both diseases are carried by rodents (family Muridae, subfamily Sigmodontinae) and each particular species of hantavirus has a particular rodent as its intermediate host. Environmental conditions that favor the reproduction and spread of rodents in endemic areas are known to increase the incidence of the disease. Humans typically acquire the disease through the inhalation of aerosolized excreta or secreta from infected rodents.

Human-to-human transmission of the Andes virus (ANDV) was first claimed to have occurred as part of the 1996 outbreak in southern Argentina [6–8], and since then a number of studies have reported more cases and suggested the involvement of this form of transmission in both Argentina and Chile [9–11]. Importantly, however, there have been no reports of human-to-human transmission from Europe, Asia, and most countries in the Americas where the disease exists, and other studies in Argentina [12, 13] and Chile [14, 15] did not find evidence of it.

It is important to determine whether there is in fact sufficient evidence for human-to-human transmission of the disease. If so, this may require recommendations regarding infection prevention and control measures in health facilities and in household settings. Thus, the objective of this systematic review was to assess the evidence for human-to-human transmission of hantavirus.

## METHODS

High-quality systematic review methods were used [16] and the protocol was registered on the International Prospective Register of Systematic Reviews (PROSPERO) [17]. Reporting of the systematic review follows the Preferred Reporting Items for Systematic Reviews and Meta-Analysis (PRISMA) statement for reporting [18].

### Search Strategy and Selection Criteria

We searched Cochrane Central, Embase, Latin American and Caribbean Health Sciences Literature (LILACS), PubMed, and Scientific Electronic Library Online (SciELO) from inception to date of search (3 July 2020). The PubMed search was updated 28 February 2021. In addition, Google was searched using the same key words for gray literature. Reference lists of included studies, key literature reviews, and Pan American Health Organization/World Health Organization documents, such as country-level guidelines, were scanned for relevant studies. Google Scholar was used to search for articles that cite key articles. Contact was made with known hantavirus experts to identify both published and unpublished studies, though the response was poor, possibly due to the fact that many of the key studies were published >20 years ago.

The search terms included medical subject heading (MeSH) terms (where relevant for the database) and text words. Searches were conducted by 1 review author (M. M. H.) and references were imported into Endnote. The search strategy and results for each of the databases are included in Supplementary File 1.

The screening of the titles and abstracts against the inclusion criteria (Table 1) was conducted by 2 review authors independently (M. M. H. and L. S. L.). The full text of any potentially relevant papers identified by *either* reviewer was retrieved and assessed against the inclusion criteria by 2 reviewers (M. M. H. and L. S. L.) independently. Disagreements regarding eligibility of studies were resolved via discussion and consensus, with consultation with a third reviewer if needed (J. T./L. R.).

**Table 1.** Eligibility Criteria for Inclusion of Studies

Element	Inclusion Criteria^a^
Participants	Studies focused on individuals (humans) of any age with a laboratory diagnosis of hantavirus infection and people in contact with them, including healthcare workers.
Exposure	Interpersonal contact with a person with laboratory-confirmed hantavirus infection within the incubation period from exposure to illness onset. Studies that only focus on human contact with infected rodents were excluded.
Comparisons	No exposure to a human with a laboratory diagnosis of hantavirus infection; low level of exposure; exposure only to infected rodents; or no comparison.
Outcomes	Transmission of hantavirus from 1 human to another. Studies were included if they had at least 1 of the following transmission outcomes measured in human contacts: • Genetic sequence analysis of the hantavirus RNA between human index and contact cases, showing homology OR • Laboratory diagnosis of hantavirus infection, including 1 of virology, serology, or immunohistochemistry OR • Signs and symptoms consistent with the clinical case definition of hantavirus pulmonary syndrome or hemorrhagic fever with renal syndrome (as defined by the country in which the study was conducted).
Study type	The evidence was prioritized by strength of study design as follows: (1) randomized controlled trials; (2) nonrandomized comparative studies (controlled trials, cohort studies, case-control studies, and cross-sectional studies); (3) noncomparative studies (eg, an epidemiological investigation of hantavirus clusters, case series, seroepidemiological studies of exposed populations only). Single case reports, modeling studies, animal-only studies, and environmental studies (ie, with no measurement of humans) were excluded. Literature reviews on the topic were also excluded but the reference list was checked for possible studies.

^a^Other criteria: Both published and unpublished (gray literature) studies in any language were included, with no date of publication limitations.

### Data Extraction, Risk of Bias Assessment, and Data Analysis

Two reviewers independently extracted all relevant data from the included papers (M. M. H. and L. S. L.) into a Microsoft Excel spreadsheet. Differences were resolved by discussion and consensus, with consultation with a third reviewer (J. T./L. R.) if needed.

Data extracted included study ID, year of study, country, region/city, setting of study, hantavirus species, mouse species, study type, type of outbreak, participants (number, age, sex, diagnostic criteria for hantavirus infection, disease, clinical case definition for suspected and confirmed cases), exposure (definition, setting, confounders measured), outcomes, results, and other relevant information (eg, case fatality rate, possible conflicts of interest).

The risk of bias (RoB) of included studies was assessed by 1 reviewer (M. M. H.) and verified by a second (L. S. L.), with consultation with a third reviewer in the case of disagreements (J. T./L. R.). The Risk of Bias in Non-randomized Studies—of Exposures (ROBINS-E) tool was used for nonrandomized comparative studies [19, 20]. The 7 RoB items include bias due to: (1) confounding, (2) selection of participants into the study, (3) classification of exposures, (4) departures from intended exposures, (5) missing data, (6) measurement of outcomes, and (7) selection of reported results. Judgments for each RoB item and for the overall judgement of the study can be low RoB, moderate RoB, serious RoB, or critical RoB [19]. No suitable tool for assessing noncomparative studies could be found in the literature and similar systematic reviews of human-to-human or animal-to-human transmission of infectious agents that we could find did not undertake a RoB assessment [21–24]. Thus, for noncomparative studies that met our inclusion criteria, no RoB assessment was undertaken. However, given the lack of a control group, the high possibility of selection bias, and inability to control for confounding, these studies are considered to be at critical RoB.

Data were synthesized in both tabular and narrative formats. A meta-analysis was not possible due to heterogeneity in the studies and outcomes measured.

## RESULTS

**Figure 1.**
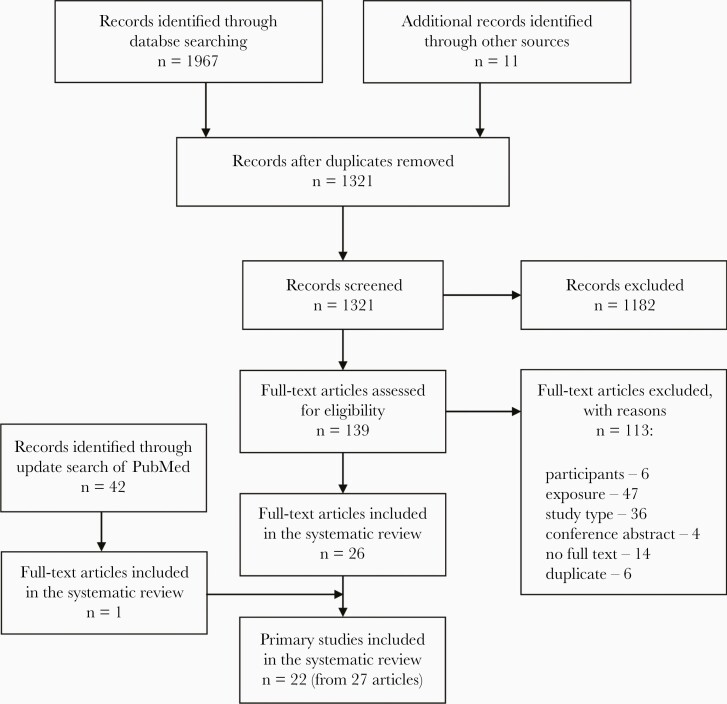
Study selection flow diagram - human-to-human transmission of hantavirus.

We identified 1321 records after removal of duplicates (Figure 1). The initial search resulted in the inclusion of 26 articles and the updated search of PubMed identified 1 additional article [25]. Thus, we included 22 primary studies in the systematic review reported in 21 articles [8–15, 25–38] and 6 supporting references 6, 7, 28, 39–41. The most common reasons for exclusion of studies at the full-text stage were due to the exposure not being interpersonal contact (n = 47) or study type (n = 36) (Figure 1 and Supplementary File 2).

### Characteristics of Included Studies

No randomized controlled trials were found. The studies included a total of 5190 participants: 4179 in the comparative designs (Table 2) and 1011 in the noncomparative designs (Supplementary Table 1). The majority of studies were conducted in Central and South America (n = 16), including 8 in Argentina and 5 in Chile. Only 8 studies used a comparative design [12, 15, 27, 29, 34, 36, 38], with 6 of these being cross-sectional serological studies [12, 15, 27, 36, 38], 1 a cohort study [29], and 1 combining both a cross-sectional and cohort design [34]. The remaining 14 studies used noncomparative designs, 10 of which involved the investigation of 1 or more clusters of cases [8–11, 25, 26, 32, 33, 35, 37], 2 were serological surveys of contacts [13, 14], and 2 investigated pregnant women with hantavirus infection for the possibility of vertical transmission of infection to their babies [30, 31].

**Table 2.** Characteristics of Included Studies: Comparative Designs

Study^a,b^ [Reference]	Country, Year of Study	Virus	Study Type	Participants	Exposure Definition (or Actual)^c^	Outcomes Measured^d^
Bayard 2004a [27]	Panama 2000	Choclo	Cross-sectional seroprevalence and epidemiological study among household and neighborhood members of index HPS patients	N = 311 (age range 1–79 years, 57% male)	Household contact (n = 10)	Serology: IgG; plus IgM in those positive for IgG
Bayard 2004b [27]	Panama 2000	Choclo	Cross-sectional seroprevalence and epidemiological study among HCW contacts of index cases compared to an unexposed group of HCWs from another department within the hospital.	N = 77; 38 exposed and 39 unexposed	Healthcare contact: medical staff who provided direct medical care (ie, <1 m from the patient) to infected patients in the emergency room and intensive care unit.	Serology: IgG; plus IgM in those positive for IgG
Chaparro 1998 [15]	Chile 1997	Andes	Cross-sectional seroprevalence and epidemiological study of HCW in the Coyhaique Regional Hospital, where the majority of HPS patients were admitted.	N = 319; 140 exposed and 179 unexposed	Healthcare contact: delivering clinical care or administrative or housekeeping services within 2 m of a patient with HPS, working with laboratory specimens from patients with HPS, or participating in an autopsy of a patient with HPS.	Serology: IgM and IgG
Ferres 2007 [29]	Chile 2001–2005	Andes	Prospective cohort study of recent household contacts of persons with HPS. Data collection included a clinical evaluation, blood samples, and a questionnaire at baseline. The clinical evaluation and blood samples were repeated weekly for 28 days. The comparison was degree and type of contact with index case.	N = 552; 76 index cases (age range 0–77 years, 74% male); 16 household contact cases (age range 2–67 years; 37.5% male); 460 seronegative household contacts (age range 2–94 years, 51.7% male)	Household contacts >2 years of age who had resided in the same house for at least 1 night at any point from 30 days before to 7 days after the onset of symptoms in the index case patient.	Virology: RT-PCR Serology: IgM and IgG
Pini 2003 [12]	Argentina 2000	?	Cross-sectional seroprevalence and epidemiologic survey of residents of the area, in conjunction with a serologic study in rodents.	N = 341 (all ages, 44% male)	ND. Actual: responded yes to a survey question about previous contact with a confirmed HPS patient.	Serology: IgG
Ruo 1994 [34]	China 1987–1988	Hantaan & Seoul	Cross-sectional seroprevalence study in April 1987. Follow-up in April 1988 of all seronegative residents from this group (cohort): serology and questionnaire regarding their activities within the prior year. Rodent studies in 1987 to ascertain the species.	N = 1811 in 1987 (all ages >1 year, 51.2% male). In 1988, 1325 of 1592 hantavirus antibody–negative residents in 1987 were revisited.	ND. Actual: responded yes to a survey question about caring for others with HFRS.	Serology: IgG
Vitek 1996 [36]	United States 1993	Sin Nombre virus	Cross-sectional serological and epidemiological study of HCWs in 4 institutions that cared for HPS patients, processed their laboratory specimens, or conducted autopsies compared to those unexposed to HPS cases.	N = 396 HCWs (age range 16–67 years, 25.4% male)	Healthcare contact: delivering clinical care or housekeeping services within 5 ft (1.5 m) of a patient with HPS, working with laboratory specimens from patients with HPS, or participating in an autopsy on a patient with HPS.	Signs and symptoms; Serology: IgM and IgG
Williams 1997 [38]	Paraguay 1995–1996	Sin Nombre virus	Cross-sectional epidemiological investigation, including review of medical records, serosurvey, environmental evaluation and rodent trapping following an outbreak (July 1995 to January 1996). Comparison of household contacts with area residents.	N = 372; 27 household contacts and 345 area residents (age range 7–75 years; 54% male)	ND. Actual: household contact.	Serology: IgM and IgG

Abbreviations: HCW, healthcare worker; HFRS, hemorrhagic fever with renal syndrome; HPS, hantavirus pulmonary syndrome; IgG, immunoglobulin G; IgM, immunoglobulin M; ND, not defined; RT-PCR, reverse-transcription polymerase chain reaction.

^a^If a study had >1 reference, we awarded 1 reference the status of primary reference.

^b^No conflicts of interest were apparent in any of the studies.

^c^For all studies, the comparison was no exposure or less exposure.

^d^None of the studies conducted genetic sequencing.

### Risk of Bias in Included Studies (Comparative Designs Only)

Of the 8 comparative study designs, 6 were classified as having a critical RoB [12, 15, 27, 36, 38] and 2 as serious RoB [29, 34] (Table 3; Supplementary File 3). The most serious issues influencing the RoB assessment were bias due to confounding and bias due to classification of the exposure.

**Table 3.** Summary of the Risk of Bias Assessment

	Bayard 2004a [27]	Bayard 2004b [27]	Chaparro 1998 [15]	Ferres 2007 [29]	Pini 2003 [12]	Ruo 1994 [34]	Vitek 1996 [36]	Williams 1997 [38]
Domains								
Confounding	Critical	Critical	Critical	Serious	Critical	Serious	Critical	Critical
Selection	Serious	Serious	Low	Moderate	Moderate	Moderate	Moderate	Critical
Measurement of exposures	Critical	Critical	Critical	Moderate	Critical	Serious	Critical	Critical
Departures from exposures	Serious	Serious	Serious	Moderate	Serious	Serious	Serious	Serious
Missing data	Moderate	Serious	Moderate	Moderate	Moderate	Serious	Low	Moderate
Measurement of outcomes	Moderate	Moderate	Low	Low	Low	Low	Moderate	Low
Reported results	Moderate	Low	Low	Low	Low	Low	Low	Low
Overall bias	Critical	Critical	Critical	Serious	Critical	Serious	Critical	Critical

### Evidence for Human-to-Human Transmission From the Comparative Designs

Three of the 8 comparative designs were conducted in Argentina [12] or Chile [15, 29], of which 1 cohort study conducted in Chile but with a serious RoB showed increased risk of human-to-human transmission of ANDV in household contacts for sex partners and with exposure to saliva (deep kissing) [29] (Table 4). Of 476 household contacts, only 16 (3.4%) developed HPS and seropositivity. The remaining 460 (96.6%) were seronegative. Only 3 of the 16 were claimed to be definite human-to-human transmission because co-exposure to rodents was unlikely. Another 9 were probable for human-to-human transmission. Unfortunately, an unexposed group was not included in the study [29]. The other 2 studies showed no evidence for human-to-human transmission. The first, a cross-sectional study of healthcare workers in the Coyhaique Regional hospital, southern Chile, that coincided with an outbreak and where the majority of HPS patients were admitted did not show any difference in prevalence of immunoglobulin G (IgG) to hantavirus between those exposed compared to those unexposed (4.3% vs 3.4%, *P* = .66) [15]. The second, a cross-sectional study of residents of Yuto, Jujuy Province in northern Argentina (specific hantavirus not clear), did not find any difference in prevalence of IgG to hantavirus in those with previous contact with a known HPS patient compared to those without previous contact (6.1% vs 6.6%; χ ^2^ = 0.03, *P* = .86) [12]. While the hantavirus species was not clearly identified in this study, it may be Laguna Negra, Bermejo, or Orán virus rather than ANDV [42]. The 5 comparative designs conducted in Panama [27], Paraguay [38], China [34], and the United States [36] showed no evidence for increased prevalence of IgG and/or immunoglobulin M to hantavirus between those exposed and unexposed.

**Table 4.** Results: Comparative Designs

Study^a^ [Reference]	Confounders Measured	Results
Bayard 2004a [27]	Co-exposure to infected rodents	No evidence for human-to-human transmission. Two of 10 household members of confirmed patients had IgG antibodies (20%); 38 of 301 neighborhood residents had IgG antibodies (12.6%).
Bayard 2004b [27]	Co-exposure to infected rodents, use of PPE	No evidence for human-to-human transmission. No IgM antibodies were present in the 77 healthcare workers. Only 1 of 38 exposed workers had IgG antibodies: 1/38 (2.63%) vs 1/39 (2.56%) of unexposed workers. Use of PPE was variable. Co-exposure to rodents is uncertain.
Chaparro 1998 [15]	Co-exposure to infected rodents	No evidence for human-to-human transmission. None of the HCWs had evidence of recent hantavirus infection, as measured by the presence of IgM hantavirus antibodies. Twelve (3.8%) had IgG hantavirus antibodies interpreted as evidence of past hantavirus infection. There was no difference in the percentage with IgG antibodies between those exposed (6/140 [4.3%]) compared to those unexposed (6/179 [3.4%]), *P* = .66. Of the exposed participants, 5 (3.6%) experienced at least 1 febrile respiratory illness episode requiring hospitalization during their lifetime vs 12 (6.7%) among those unexposed (χ ^2^, 1 *df* = 1.53; *P* = .21). Use of PPE was variable.
Ferres 2007 [29]	Co-exposure to infected rodents	Of 476 household contacts, 16 (3.4%) developed HPS; 32.6% of the 92 cases occurred in 14 household clusters. 460/476 contacts remained seronegative. The risk of HCPS was 17.6% among sex partners of index case patients vs 1.2% among other household contacts (*P* < .001). For sex partners: adjusted OR, 9.71 (95% CI, 1.72–54.67). For exposure to saliva (deep kissing): adjusted OR, 5.05 (95% CI, .89–28.52). Human-to-human transmission appeared definite in 3 (ie, no environmental co-exposure, urban residence), probable in 9 (ie, close contact with the index case patient, including 7 of 9 who were sex partners, and a lack of major risk factors for environmental exposure), and possible in 2 of the 16 additional household case patients. The remaining 2 had a shared environmental exposure.
Pini 2003 [12]	Co-exposure to infected rodents	No evidence for human-to-human transmission. Hantavirus IgG was found in 22/341 (6.5%) serum samples tested. Hantavirus antibodies among persons who had previous contact with known HPS patients vs those who did not were 6/98 (6.1%) vs 16/242 (6.6%) (χ ^2^ = 0.03, *P* = .86). Rural occupation was the risk factor that showed a significant difference between antibody-positive and -negative persons with 20/201 (10%) IgG positive vs 1/97 (1.03%) for urban or suburban occupations (χ ^2^ = 7.95, *P* = .004). No hantavirus antibodies were found among 61 students or 20 healthcare workers, including physicians, nurses, health agents, and a dentist. The presence of rodents was reported by 77% of hantavirus antibody–positive and 79% of hantavirus antibody–negative persons (χ ^2^, *P* > .05), both in peridomestic and workplace settings. 28.7% reported contact with a confirmed HPS case patient.
Ruo 1994 [34]	Co-exposure to infected rodents	No evidence for human-to-human transmission. Family clustering was analyzed by binomial distribution fit procedure in the cross-sectional sample in 1987; no clustering was found (χ ^2^ = 0.59, 1 *df*, *P* = .44). Thirty of 1325 (2.3%) residents who were IgG-seronegative in 1987 seroconverted between 1987 and 1988. A multivariate analysis of risk factors in each of the cross-sectional and cohort studies found a significant risk for 3–4 factors associated with rodent exposure. Caring for others with HFRS did not show a significant relationship with IgG positivity in the univariate analysis (data not shown) and was not included in the multivariate analysis.
Vitek 1996 [36]	PPE	No evidence for human-to-human transmission. None of the 396 participants had serological evidence of recent or past hantavirus infection. There were no statistically significant differences between exposed and unexposed HCWs in the percentage reporting any symptoms of illness since 1 April 1993. Use of some form of PPE was high (eg, 82% reported using gloves and 80% reported using some type of mask [but no mention of characteristics] in all their encounters with HPS patients). 70.6% reported exposure to patients with HPS.
Williams 1997 [38]	Co-exposure to infected rodents	No evidence for human-to-human transmission. Forty-four of 345 (12.8%) area residents included in the convenience sample and 4/27 (14.8%) household contacts had IgG antibodies to SNV antigen. All 4 of the SNV antibody-positive household contacts were linked to case #1, which had evidence of rodent exposure at home. Six of the 78 (7.7%) tested rodents had IgG antibodies to SNV antigen.

Abbreviations: CI, confidence interval; *df*, degrees of freedom; HCW, healthcare worker; HPS, hantavirus pulmonary syndrome; IgG, immunoglobulin G; IgM, immunoglobulin M; OR, odds ratio; PPE, personal protective equipment; SNV, Sin Nombre virus.

^a^If a study had >1 reference, we awarded 1 reference the status of primary reference.

### Evidence for Human-to-Human Transmission From the Noncomparative Designs

Of the 14 noncomparative studies, 7 were conducted in Argentina (6 analyses of clusters and 1 seroprevalence study) [8–10, 13, 25, 26, 32] and 3 in Chile (2 analyses of clusters and 1 seroprevalence study) [11, 14, 35] (Supplementary Table 2). None of the 4 noncomparative studies conducted outside of Argentina and Chile found evidence for human-to-human transmission [30, 31, 33, 37]. In Argentina, all but 1 of the studies showed some evidence of human-to-human transmission in investigations of clusters [8–10, 25, 26, 32], with up to 16 likely cases of human-to-human transmission reported between 1996 and 2014. The number of likely cases of human-to-human transmission from the 2018–2019 outbreak in Chubut province of Argentina cannot be determined due to lack of detailed epidemiological and environmental investigation [25]. While the authors claim that there were 33 cases of human-to-human transmission and genetic sequencing showing 99.8%–100% identity of the ANDV sequences between cases, the identical sequences can also be explained by exposure to the same viral variant within the local rodent populations [10, 32] (most of the cases lived in the same town), and co-exposure to rodents cannot be ruled out. Furthermore, some of the claimed events had minimal contact with a known case. The genetic sequencing conducted as part of 5 of the studies of clusters supported human-to-human contact in a maximum of 12 cases (2 for Iglesias et al [9], 2 for Martínez et al [32], 2 for Wells et al [8], and up to 6 for Martínez et al [25]—but with the limitation of no investigation of rodent exposure) due to identical sequences but likely different geographic area of infection. The seroprevalence study conducted 1–2 months following the 1996 outbreak in Rio Negro province did not support the finding of human-to-human transmission [13]. It found a very low prevalence of IgG to hantavirus (1%) in 294 community members and 0% in 152 healthcare workers [13]. Interestingly, the authors of this article make reference to a case-control study conducted as part of the outbreak, but we could not find any further details in the published literature, nor from the authors contacted.

Similar to the case in Argentina, the 2 studies investigating clusters in Chile did find some evidence of human-to-human transmission, with up to 5 likely cases of human-to-human transmission reported [11, 35]. However, the seroprevalence study conducted in Temuco, Chile, did not support the finding of human-to-human transmission [14]. It found a prevalence of IgG to hantavirus of 1.9% in 106 family contacts and 0% in 109 healthcare worker contacts, which are both lower than the seroprevalence of 2.5%–7.5% in the rural communities where they live [14].

## DISCUSSION

This systematic review presents a comprehensive and systematic evaluation of the research on human-to-human transmission of hantavirus. Despite claims of authors from Argentina and Chile of the existence of human-to-human transmission of ANDV, the balance of the evidence does not support this claim. Evidence from comparative studies (the strongest level of evidence available) does not support human-to-human transmission of hantavirus infection [12, 15, 27, 34, 36, 38], with the exception of 1 prospective cohort study in Chile with serious RoB and no unexposed comparison group [29]. For the noncomparative studies, the evidence for human-to-human transmission is limited to analyses of clusters in Chile [11, 35] and Argentina [8–10, 26, 32] and these findings are not supported by seroprevalence studies conducted in these countries after hantavirus outbreaks in the population [13, 14]. By design, noncomparative studies are severely limited in their power to make causal inferences due to common selection biases and confounding [43, 44].

The absolute number of cases that may be attributable to human-to-human transmission is low and needs to be contrasted with the much larger number of cases that did not lead to human-to-human transmission, even with similarly close contact in community or health facility settings. For example, the cohort study by Ferres and colleagues included 476 household contacts, of which only 16 subsequently developed HPS, 3 of which were claimed to be definitely due to human-to-human transmission and 9 probably due to human-to-human transmission; the remaining 460 household contacts did not [29]. Their multivariate logistic regression model showed that the odds of infection among contacts were increased for sex partners and with exposure to saliva (deep kissing). However, the possible confounding effect of environmental exposure to the excreta/secreta of infected rodents, which is a known cause of infection, was not considered in this model, though it was considered qualitatively [29].

Analysis of the evidence from noncomparative studies suggests a possibility of human-to-human transmission of ANDV in some parts of Argentina and Chile. However, as noted above, these studies are not able to make causal inferences and are limited to a few outbreaks, and the findings of some outbreaks have been published across different reports, with some inconsistencies in the accounts (see, eg, Supplementary File 4 [6–8, 28]). Furthermore, despite the known limitations of investigations of cases (without any suitable control group), some authors have made some rather alarming claims, such as the existence of “super-spreaders,” a term that is currently in vogue due to the coronavirus disease 2019 pandemic but was also used for outbreaks caused by Ebola virus, Middle East respiratory syndrome coronavirus, and severe acute respiratory syndrome coronavirus—all viruses that are proven to be caused by human-to-human transmission and with combined modes of transmission [25]; this is not the case for hantavirus in the face of the current knowledge and available data. The authors of the “super-spreaders” article investigated a cluster of 34 cases and claim that all 33 cases resulting from the index case were caused by human-to-human transmission, but do not consider alternative explanations for the cases. No investigation of possible co-exposure to the excreta/secreta of infected rodents in the environment seems to have been conducted and the possibility of environmental exposure is not even considered as a possible limitation of the study. Moreover, despite the finding of Ferres et al in Chile that infection due to interpersonal contact is rare and was only found among close relatives in their study (9 of which were married or cohabiting couples) [29], Martínez and colleagues even suggest that crossing paths on the way to the restroom at a party without any physical contact was enough to result in transmission (as was the case for patient 4 with the index case; see Figure 1*B* and Supplementary Figure 3 in Martínez et al [25]). Yet, there is no discussion as to why the person sitting next to the febrile index case did not become infected, nor the other ≥6 people sitting less than a meter away from him at the party.

While the use of genetic sequencing data to confirm human-to-human transmission has been used in investigations of clusters in Argentina and Chile [7–11, 25, 26, 32, 35], it has the limitation of only providing strong evidence when cases that occur in different geographic areas have identical sequences. “When all case-patients remain in the disease endemic area … molecular dissimilarities rule out person-to-person transmission, but identical sequences do not support it” [10].

A key strength of this review was the use of high-quality systematic review methods [16] that included the use of 2 reviewers for all methodological stages of the review and a comprehensive search strategy. However, this review is limited by the study designs and high RoB of all but 1 of the included studies. Furthermore, the lack of response from most of the authors of the included studies limited our ability to check methodological issues or to determine why the case-control study referred to in conjunction with the 1996 Rio Negro outbreak in El Bolsón and Bariloche was never published [13].

The RoB in included studies is a particular concern when trying to determine whether human-to-human transmission of hantavirus is the best explanation for infection in other human contacts. A randomized controlled trial, where subjects are randomized to exposure to another human with hantavirus or to a nonexposure control group, in theory, would constitute the ideal study design. However, ethical and feasibility concerns make this kind of study impractical at this stage of our understanding of the problem. The next best study designs fall in the observational design category but also require the use of a control (or comparison) group, such as in a cohort or case-control design—which are commonly used designs for outbreak investigations [44]. These would need to include the conduct of a multivariate analysis to control for potential confounders, especially environmental exposure to the excreta/secreta of infected rodents and the correct and consistent use of personal protective equipment (for studies of healthcare workers). The possibility of confounding due to co-exposure of the contact to the excreta/secreta of rodents with hantavirus and bias due to measurement of the exposure (interpersonal contact with a person with hantavirus infection) require particular attention for this research question. Furthermore, all but 1 of the comparative studies [29] had serious risk of differential misclassification of the exposure due to limited information about the duration, type or time of contact with the infected person, and environmental co-exposure to the excreta/secreta of infected rodents. Thus, the investigation of cases and contacts should include the characterization of the type of activity during the interpersonal contact (including the possibility of exposure to bodily fluids or aerosolized virus particles) and the duration and frequency of the interpersonal contact. In addition, environmental investigation of all places frequented by each case and contact (workplace, home, leisure) should be undertaken.

What are the public health implications of these findings? Given the high case fatality rate for HPS, the precautionary principle could be invoked to recommend action to implement infection prevention control containment measures in cases of suspected hantavirus infection in health facilities and household settings where ANDV is present. This could include the implementation of standard precautions and rational and optimized use of personal protective equipment, including the use of filtering facepiece respirators, or respirators by health workers to prevent possible infection by inhalation of droplets or aerosolized virions. However, given the limited number of cases and the high RoB in included studies, it may be prudent to accompany such a recommendation with a strategy to gather better research evidence to answer the question.

Future studies should include a control group, such as in a cohort or case-control design, and conduct a multivariate analysis to control for potential confounders, especially factors related to the potential risk of environmental exposure to the excreta/secreta of infected rodents. Care should be taken to measure the duration, type, and timing of contact with the infected person and with infected rodents. Outcome measures should include laboratory measures such as virology, serology, or immunohistochemistry. Molecular epidemiology analysis, including genetic sequencing of rodent and human specimens, may also be useful when cases have occurred outside the affected geographic area.

In conclusion, this systematic review has shown that the evidence for human-to-human transmission of hantavirus is weak, specific to ANDV, and limited to some parts of Argentina and Chile. Due to the high case fatality rate for HPS, it may be prudent to recommend infection prevention and control measures in cases of suspected hantavirus infection—but this needs to be accompanied by the gathering of better research evidence.

## Supplementary Data

Supplementary materials are available at *The Journal of Infectious Diseases* online. Supplementary materials consist of data provided by the author that are published to benefit the reader. The posted materials are not copyedited. The contents of all supplementary data are the sole responsibility of the authors. Questions or messages regarding errors should be addressed to the author.

Supplementary File 1. Search terms and results

Supplementary File 2. List of excluded studies

Supplementary File 3. Risk of bias assessment

Supplementary File 4. Description of HPS cases, Rio Negro Province, Argentina 1996

Supplementary Table 1. Characteristics of included studies—noncomparative designs by country of study

Supplementary Table 2. Results—noncomparative designs by country of study

jiab461_suppl_Supplementary_Materials

## Notes

***Author contributions.*** J. T. and L. R. conceptualized the study. M. M. H., with significant input from J. T., L. R., and L. S. L., wrote the study protocol. M. M. H. and L. S. L. assessed eligibility, extracted data, evaluated the risk of bias, and undertook the narrative synthesis. J. T. and L. R. provided input in the case of disagreements or doubts. M. M. H. wrote the first draft of the manuscript with significant input from L. S. L., J. T., R. A., and L. R. All authors provided conceptual input and revised and approved the final version of the manuscript. M. M. H. and L. S. L. have verified the underlying data. The data were available to all authors on request. J. T. and M. M. H. had final responsibility for the decision to submit for publication.

***Acknowledgments.*** This review was funded by the Pan American Health Organization (PAHO)/World Health Organization (WHO). We thank Rosemeire Pinto (Biblioteca Regional de Medicina librarian) for assistance with the search strategies.

***Disclaimer.*** Some authors are staff members of American Health Organization (PAHO)/World Health Organization (WHO). Authors hold sole responsibility for the views expressed in the manuscript, which may not necessarily reflect the opinion or policy of PAHO/WHO. Apart from the participation of the named PAHO/WHO authors, the funder of the study had no role in study design, data collection, data analysis, data interpretation, or writing of the report.

***Financial support.*** This work was supported by PAHO through the cooperative grant award NU50CK000494, “Building Capacity and Networks to Address Emerging Infectious Diseases in the Americas,” from the Centers for Disease Control and Prevention.
