# Supplementary material for: Evidence for Human-to-Human Transmission of Hantavirus: A Systematic Review
Source: J Infect Dis. 2021 Sep 13;226(8):1362–71. doi: 10.1093/infdis/jiab461 (PMC9574657; doi:10.1093/infdis/jiab461)
Supplement: jiab461_suppl_Supplementary_Materials [file jiab461_suppl_supplementary_materials.docx]

**SUPPLEMENTARY MATERIALS FOR**

“Evidence for human-to-human transmission of hantavirus: a systematic review”

**TABLE OF CONTENTS**

[**Supplementary File 1:** Search terms and results 2](#_Toc78298974)

[**Supplementary File 2:** List of excluded studies 6](#_Toc78298975)

[**Supplementary File 3:** Risk of bias assessment 14](#_Toc78298976)

[**Supplementary File 4:** Description of HPS cases, Rio Negro Province, Argentina 1996 17](#_Toc78298977)

[**Supplementary Table 1:** Characteristics of included studies – non-comparative designs
by country of study 22](#_Toc78298978)

[**Supplementary Table 2:** Results – non-comparative designs by country of study 25](#_Toc78298979)

[**References** 29](#_Toc78298980)

# **Supplementary File 1:** Search terms and results

## **Search terms used when searching for studies – MESH terms, title, abstract and keywords**

**1. Hantavirus**

(hantavirus* OR "Dobrava Belgrade virus" OR "Andes Hantavirus" OR "Andes Hantaviruses" OR "hantavirus pulmonary syndrome" OR "hantavirus associated respiratory distress syndrome" OR "hantavirus infection" OR "hantavirus Infections" OR "Hemorrhagic Fever with Renal Syndrome") OR HARDS[tiab]

Spanish/Portuguese terms:

"Virus Dobrava Belgrado" OR "Vírus Dobrava-Belgrado" OR "Hantavirus de los Andes" OR "Síndrome Pulmonar por Hantavirus" OR "Síndrome Pulmonar por Hantavírus" OR "Infecciones por Hantavirus" OR "Infeccion por Hantavirus" OR "Infecções por Hantavirus" OR "febre hemorrágica com síndrome renal" OR "fiebre hemorrágica con síndrome renal"

**2. Transmission**

("autochthonous transmission" OR "communicable disease transmission" OR "community-acquired infections" OR "cross infection" OR ("patient professional" AND transmission) OR "healthcare associated infection" OR "communicable diseases transmission" OR "community-acquired infection" OR "cross infections" OR "healthcare associated infections" OR "hospital infection" OR "infection control" OR "infection transmission" OR "infectious disease transmission" OR "nosocomial infection" OR "pathogen transmission" OR "patient isolation" OR "diseases transmission" OR "Infectious Diseases Transmission" OR ("horizontal transmission" AND infectious disease*) OR "hospital infections" OR "infections control" OR "infections transmission" OR "nosocomial infections" OR "pathogens transmission" OR "patients isolation" OR "disease transmission" OR Transmission[MeSH Subheading] OR contagio*[tiab] OR transmission[tiab] OR transmited*[tiab] OR communicable[tiab] OR transmit*[tiab])

Spanish/Portuguese terms:

"infeccion hospitalaria" OR "infecção hospitalar" OR "control de infecciones" OR "controle de infecções" OR "transmisión de enfermedad infecciosa de profesional a paciente" OR "transmissão de doença infecciosa do profissional para o paciente"

**3. Human**

**human[tiab] OR humans[tiab] OR humans[mh]**

Variations of the search terms for hantavirus and transmission were also searched in Spanish and Portuguese – where the search engine supported foreign characters.

Keyword areas were combined using AND

## **Search results for each source – Human-to-human transmission of hantavirus**

| **Database** | **Keyword areas searched** | **Date searched** | **No. refs found** | **No. refs after duplicates removed** | **Potentially meets the inclusion criteria** | **Incl.** |
| --- | --- | --- | --- | --- | --- | --- |
| **Electronic Databases** |  |  |  |  |  |  |
| Cochrane CENTRAL | 1 | 3/7/20 | 60 | 56 | 4 | 0 |
| Embase (Ovid) | 1, 2, 3 | 3/7/20 | 893 | 319 | 28 | 1 |
| LILACS (BVSalud) | 1, 2 | 3/7/20 | 80 | 54 | 7 | 0 |
| PubMed (NLM) | 1, 2, 3 | 3/7/20 | 875 | 870 | 86 | 23 |
| SciELO | 1, 2 | 3/7/20 | 59 | 11 | 3 | 0 |
| **Total:** |  |  | **1967** | **1310** | **128** | **24** |
| **Manual searches** |  |  |  |  |  |  |
| Google, Google Scholar (citations), reference lists of included/excluded studies, contact with authors | 1, 2 | Early August 2020 | 11 | 11 | 11 | 2 |
| **Total:** |  |  |  | **1321** | **139** | **26** |
| PubMed (NLM) update search | 1, 2, 3 | 28/2/21 | 42 |  | 4 | 1 |
| **TOTAL** |  |  |  |  |  | **27** |

## **Search strategies**

**Cochrane CENTRAL (Ovid) – 3 July 2020**

Database: EBM Reviews - Cochrane Central Register of Controlled Trials <May 2020>

Search Strategy:

--------------------------------------------------------------------------------

1 (hantavirus$ or "Dobrava Belgrade virus" or "Andes Hantavirus" or "Andes Hantaviruses" or "hantavirus pulmonary syndrome" or "hantavirus associated respiratory distress syndrome" or "hantavirus infection" or "hantavirus Infections" or "Hemorrhagic Fever with Renal Syndrome").af. (60)

2 HARDS.ti. or HARDS.ab. (0)

3 1 or 2 (60)

**EMBASE (Ovid) – 3 July 2020**

Database: Embase Classic+Embase <1947 to 2020 Week 26>

Search Strategy:

--------------------------------------------------------------------------------

1 (hantavirus$ or "Dobrava Belgrade virus" or "Andes Hantavirus" or "Andes Hantaviruses" or "hantavirus pulmonary syndrome" or "hantavirus associated respiratory distress syndrome" or "hantavirus infection" or "hantavirus Infections" or "Hemorrhagic Fever with Renal Syndrome").af. (5971)

2 HARDS.ti. or HARDS.ab. (10)

3 1 or 2 (5980)

4 ("autochthonous transmission" or "communicable disease transmission" or "community-acquired infections" or "cross infection" or ("patient professional" and transmission) or "healthcare associated infection" or "communicable diseases transmission" or "community-acquired infection" or "cross infections" or "healthcare associated infections" or "hospital infection" or "infection control" or "infection transmission" or "infectious disease transmission" or "nosocomial infection" or "pathogen transmission" or "patient isolation" or "diseases transmission" or "Infectious Diseases Transmission" or ("horizontal transmission" and infectious disease$) or "hospital infections" or "infections control" or "infections transmission" or "nosocomial infections" or "pathogens transmission" or "patients isolation" or "disease transmission").af. (276426)

5 transmission/ (7223)

6 (contagio$ or transmission or transmited$ or communicable or transmit$).ti. or (contagio$ or transmission or transmited$ or communicable or transmit$).ab. (602540)

7 4 or 5 or 6 (816484)

8 (human or humans).ti. or (human or humans).ab. (3408261)

9 human/ (22211796)

10 8 or 9 (23019285)

11 3 and 7 and 10 (893)

***************************

**LILACS (**[**https://bvsalud.org/en/**](https://bvsalud.org/en/)**) – 3 July 2020**

(tw:(hantavirus* OR "Dobrava Belgrade virus" OR "Andes Hantavirus" OR "Andes Hantaviruses" OR "hantavirus pulmonary syndrome" OR hards OR "hantavirus associated respiratory distress syndrome" OR "hantavirus infection" OR "hantavirus Infections" OR "Hemorrhagic Fever with Renal Syndrome" OR "Virus Dobrava Belgrado" OR "Vírus Dobrava-Belgrado" OR "Hantavirus de los Andes" OR "Síndrome Pulmonar por Hantavirus" OR "Síndrome Pulmonar por Hantavírus" OR "Infecciones por Hantavirus" OR "Infeccion por Hantavirus" OR "Infecções por Hantavirus" OR "febre hemorrágica com síndrome renal" OR "fiebre hemorrágica con síndrome renal"))

AND (tw:((tw:("autochthonous transmission" OR "communicable disease transmission" OR "community-acquired infections" OR "cross infection" OR ("patient professional" AND transmission) OR "healthcare associated infection" OR "communicable disease transmission" OR "community-acquired infection" OR "cross infections" OR "healthcare associated infections" OR "hospital infection" OR "infection control" OR "infection transmission" OR "infectious disease transmission" OR "infectious disease transmission" OR "nosocomial infection" OR "pathogen transmission" OR "patient isolation" OR "diseases transmission" OR "Infectious Diseases Transmission" OR ("horizontal transmission" AND infectious disease*) OR "hospital infections" OR "infections control" OR "infections transmission" OR "nosocomial infections" OR "pathogens transmission" OR "patients isolation" OR "disease transmission" OR "infeccion hospitalaria" OR "infecção hospitalar" OR "control de infecciones" OR "controle de infecções" OR "transmisión de enfermedad infecciosa de profesional a paciente" OR "transmissão de doença infecciosa do profissional para o paciente")) OR (ti:(contagio* OR transmission OR transmited* OR transmit* OR communicable)) OR (ab:(contagio* OR transmission OR transmited* OR transmit* OR communicable))))

AND (db:(“LILACS”)

N=80

**PubMED (NLM) – 3 July 2020**

| **Search** | **Query** | **Items found** |
| --- | --- | --- |
| #4 | Search #1 AND #2 AND #3 | 875 |
| #3 | Search ((human[Title/Abstract] OR humans[Title/Abstract])) OR humans[MeSH Terms] | 19157140 |
| #2 | Search ((("autochthonous transmission" OR "communicable disease transmission" OR "community-acquired infections" OR "cross infection" OR ("patient professional" AND transmission) OR "healthcare associated infection" OR "communicable diseases transmission" OR "community-acquired infection" OR "cross infections" OR "healthcare associated infections" OR "hospital infection" OR "infection control" OR "infection transmission" OR "infectious disease transmission" OR "nosocomial infection" OR "pathogen transmission" OR "patient isolation" OR "diseases transmission" OR "Infectious Diseases Transmission" OR ("horizontal transmission" AND infectious disease*) OR "hospital infections" OR "infections control" OR "infections transmission" OR "nosocomial infections" OR "pathogens transmission" OR "patients isolation" OR "disease transmission")) OR Transmission[MeSH Subheading]) OR (contagio*[Title/Abstract] OR transmission[Title/Abstract] OR transmited*[Title/Abstract] OR communicable[Title/Abstract] OR transmitt*[Title/Abstract]) | 685236 |
| #1 | Search (((hantavirus* OR "Dobrava Belgrade virus" OR "Andes Hantavirus" OR "Andes Hantaviruses" OR "hantavirus pulmonary syndrome" OR "hantavirus associated respiratory distress syndrome" OR "hantavirus infection" OR "Hemorrhagic Fever with Renal Syndrome" OR "Hantavirus Infections" OR "Hantavirus Infection"))) OR HARDS[Title/Abstract] | 5590 |

**SciELO (**[**https://scielo.org/en**](https://scielo.org/en)**) – 31 July 2018**

(hantavirus* OR "Dobrava Belgrade virus" OR "Andes Hantavirus" OR "Andes Hantaviruses" OR "hantavirus pulmonary syndrome" OR "hantavirus associated respiratory distress syndrome" OR "hantavirus infection" OR "hantavirus Infections" OR "Hemorrhagic Fever with Renal Syndrome" OR HARDS) OR ("Virus Dobrava Belgrado" OR "Vírus Dobrava-Belgrado" OR "Hantavirus de los Andes" OR "Síndrome Pulmonar por Hantavirus" OR "Síndrome Pulmonar por Hantavírus" OR "Infecciones por Hantavirus" OR "Infeccion por Hantavirus" OR "Infecções por Hantavirus" OR "febre hemorrágica com síndrome renal" OR "fiebre hemorrágica con síndrome renal")

AND

("autochthonous transmission" OR "communicable disease transmission" OR "community-acquired infections" OR "cross infection" OR ("patient professional" AND transmission) OR "healthcare associated infection" OR "communicable disease transmission" OR "community-acquired infection" OR "cross infections" OR "healthcare associated infections" OR "hospital infection" OR "infection control" OR "infection transmission" OR "infectious disease transmission" OR "infectious disease transmission" OR "nosocomial infection" OR "pathogen transmission" OR "patient isolation" OR "diseases transmission" OR "Infectious Diseases Transmission" OR ("horizontal transmission" AND infectious disease*) OR "hospital infections" OR "infections control" OR "infections transmission" OR "nosocomial infections" OR "pathogens transmission" OR "patients isolation" OR "disease transmission" OR "infeccion hospitalaria" OR "infecção hospitalar" OR "control de infecciones" OR "controle de infecções" OR "transmisión de enfermedad infecciosa de profesional a paciente" OR "transmissão de doença infecciosa do profissional para o paciente") OR (ti:(contagio* OR transmission OR transmited* OR transmit* OR communicable)) OR (ab:(contagio* OR transmission OR transmited* OR transmit* OR communicable))

N=59

# **Supplementary File 2:** List of excluded studies

Excluded studies (N=113)

93 with reason,

6 duplicates,

14 no full text

**List of excluded studies with reason for exclusion (N=93)**

Reason: participants – 6; exposure – 47; study type – 36; conference abstract only – 4

| **Reference** | **Reason for exclusion** |
| --- | --- |
| Akar N, Caliskan E, Ozturk CE, Ankarali H, Kilincel O, Oksuz S, et al. Seroprevalence of hantavirus and borrelia burgdorferi in duzce (Turkey) forest villages and the relationship with sociodemographic features. Turkish Journal of Medical Sciences 2019;49: 483-9. | Exposure - not interpersonal contact |
| Altun DU, Mehmet Ali Oktem I, Unal B. Hantavirus infections and prevention of hantaviruses. TAF Preventive Medicine Bulletin 2011;10: 373-8. | Study type - review |
| Anonymous. Hantavirus pulmonary syndrome in the Americas. Revista Panamericana de Salud Publica/Pan American Journal of Public Health 1998;3: 351-3. | Study type - review |
| Bao C-j, Guo X-l, Qi X, Hu J-l, Zhou M-h, Varma JK, et al. A Family Cluster of Infections by a Newly Recognized Bunyavirus in Eastern China, 2007: Further Evidence of Person-to-Person Transmission. Clinical Infectious Diseases 2011;53: 1208-14. | Participants - not hantavirus |
| Beeching NJ, Fletcher TE, Hill DR, Thomson GL. Travellers and viral haemorrhagic fevers: what are the risks? Int J Antimicrob Agents 2010;36 Suppl 1: S26-35. | Study type - review |
| Bellomo C, Alonso D, Coelho R, Iglesias A, Periolo N, Martinez VP. A newborn infected by Andes virus suggests novel routes of hantavirus transmission: a case report. Clin Microbiol Infect 2020;26: 130-1. | Study type - single case report |
| Bellomo CM, Nudelman J, Kwaszka R, Vazquez G, Cantoni G, Weinzettel B, et al. Geoaraohic expansion of hantavirus pulmonary syndrome in Argentina: The southernest case report. [Spanish]. Medicina 2009;69: 647-50. | Study type - single case report |
| Bergstedt Oscarsson K, Brorstad A, Baudin M, Lindberg A, Forssen A, Evander M, et al. Human Puumala hantavirus infection in northern Sweden; increased seroprevalence and association to risk and health factors. BMC infectious diseases 2016;16. | Exposure - not interpersonal contact |
| Bologna R, González S, Ruvinsky S. Brote de hantavirus en Epuyen, Chubut, Argentina. Med infant 2019;26: 53-6. | Study type - review and description of an outbreak |
| Boone I, Wagner-Wiening C, Reil D, Jacob J, Rosenfeld UM, Ulrich RG, et al. Rise in the number of notified human hantavirus infections since October 2011 in Baden-Wurttemberg, Germany. Euro Surveill 2012;17. | Exposure - not interpersonal contact |
| Butler JC, Peters CJ. Hantaviruses and hantavirus pulmonary syndrome. Clin Infect Dis 1994;19: 387-94; quiz 95. | Study type - review |
| Butler JC, Zaki SR, Khabbaz RF, Peters CJ. Hantavirus pulmonary syndrome. Infectious Diseases in Clinical Practice 1995;4: 189-93. | Study type - review |
| Celebi G, Oztoprak N, Oktem IMA, Heyman P, Lundkvist A, Wahlstrom M, et al. Dynamics of Puumala hantavirus outbreak in Black Sea Region, Turkey. Zoonoses Public Health 2019;66: 783-97. | Exposure - not interpersonal contact |
| Centers for Disease Control and Prevention. From the Centers for Disease Control and Prevention. Update: outbreak of hantavirus infection--southwestern United States, 1993. Jama 1993;270: 306. | Exposure - not interpersonal contact |
| Centers for Disease Control and Prevention. Hantavirus pulmonary syndrome -- Chile, 1997. MMWR Morb Mortal Wkly Rep 1997;46: 949-51. | Study type - brief report / not enough information to include |
| Chapman LE, Ellis BA, Koster FT, Sotir M, Ksiazek TG, Mertz GJ, et al. Discriminators between hantavirus-infected and -uninfected persons enrolled in a trial of intravenous ribavirin for presumptive hantavirus pulmonary syndrome. Clinical infectious diseases 2002;34: 293-304. | Exposure - not interpersonal contact |
| Chertcoff J, Quadrelli S. Hantavirus cardiopulmonary syndrome. Clinical Pulmonary Medicine 2002;9: 75-80. | Study type - review |
| Chile - Ministerio de Salud. Control y prevención de la infección por hantavirus para equipos de salud. Santiago, 2013 Diciembre 12. | Study type - clinical practice guideline / report |
| Ciancaglini M, Bellomo CM, Torres Cabreros CL, Alonso D, Bassi SC, Iglesias AA, et al. Hantavirus pulmonary syndrome in Tucuman province associated to an unexpected viral genotype. Medicina (B Aires) 2017;77: 81-4. | Study type - single case report |
| da Cruz Lamas C, de Oliveira R, Gomes da Silva R, Bassan Vicente LH, Barros de Almeida E, Sampaio de Lemos ER, et al. Hantavirus infection in HIV positive individuals in Rio de Janeiro, Brazil: A seroprevalence study. Brazilian Journal of Infectious Diseases 2013;17: 120-1. | Exposure - not interpersonal contact |
| de Borba L, Delfraro A, Raboni SM, dos Santos CN. First evidence of asymptomatic infection related to the Araucaria (Juquitiba-like) hantavirus. BMJ Case Rep 2013;2013. | Study type - single case report |
| de Figueiredo GG, Borges AA, Campos GM, Machado AM, Saggioro FP, Sabino Junior GS, et al. Diagnosis of hantavirus infection in humans and rodents in Ribeirao Preto, State of Sao Paulo, Brazil. [Portuguese]. Revista da Sociedade Brasileira de Medicina Tropical 2010;43: 348-54. | Exposure - not interpersonal contact |
| de St Maurice A, Ervin E, Schumacher M, Yaglom H, VinHatton E, Melman S, et al. Exposure Characteristics of Hantavirus Pulmonary Syndrome Patients, United States, 1993-2015. Emerg Infect Dis 2017;23: 733-9. | Exposure - not interpersonal contact |
| Desmyter J, LeDuc JW, Johnson KM, Brasseur F, Deckers C, van Ypersele de Strihou C. Laboratory rat associated outbreak of haemorrhagic fever with renal syndrome due to Hantaan-like virus in Belgium. Lancet 1983;2: 1445-8. | Exposure - not interpersonal contact |
| Diglisic G, Xiao SY, Gligic A, Obradovic M, Stojanovic R, Velimirovic D, et al. Isolation of a Puumala-like virus from Mus musculus captured in Yugoslavia and its association with severe hemorrhagic fever with renal syndrome. J Infect Dis 1994;169: 204-7. | Exposure - not interpersonal contact |
| Dixon KE, Nang RN, Kim DH, Hwang YJ, Park JW, Huh JW, et al. A hospital-based, case-control study of risk factors for hemorrhagic fever with renal syndrome in soldiers of the Armed Forces of the Republic of Korea. American Journal of Tropical Medicine and Hygiene 1996;54: 284-8. | Exposure - not interpersonal contact |
| Dusi Rde M, Bredt A, Freitas DR, Bofill MI, Silva JA, Oliveira SV, et al. Ten years of a hantavirus disease emergency in the Federal District, Brazil. Rev Soc Bras Med Trop 2016;49: 34-40. | Exposure - not interpersonal contact |
| Enria DAM, Levis SC. Emerging viral zoonoses: Hantavirus infections. [Spanish]. OIE Revue Scientifique et Technique 2004;23: 595-611. | Study type - review |
| Escalera-Antezana JP, Torrez-Fernandez R, Montalvan-Plata D, Montenegro-Narvaez CM, Aviles-Sarmiento JL, Alvarado-Arnez LE, et al. Orthohantavirus pulmonary syndrome in Santa Cruz and Tarija, Bolivia, 2018. Int J Infect Dis 2020;90: 145-50. | Exposure - not interpersonal contact |
| Fabbri M, Maslow MJ. Hantavirus pulmonary syndrome in the United States. Current Infectious Disease Reports 2001;3: 258-65. | Study type - review |
| Felices V, Aguayo N, Laguna-Torres VA, Aguilar PV, Cruz C, Allende I, et al. Laguna negra virus associated with human illness in Paraguay. American Journal of Tropical Medicine and Hygiene 2009;81: 287. | Abstract only and no full report could be found to assess the study against the inclusion criteria |
| Ferres GM, Sandoval CC, Delgado BI, Sotomayor PV, Olea NA, Vial CPA. Hantavirosis: clinical and epidemiological characteristics of pediatric patients in Chile. [Spanish]. Revista chilena de infectologia : organo oficial de la Sociedad Chilena de Infectologia 2010;27: 52-9. | Participants - no laboratory diagnosis |
| Ferres M, Martinez-Valdebenito C, Angulo J, Henriquez C, Vera-Otarola J, Vergara MJ, et al. Mother-to-Child Transmission of Andes Virus through Breast Milk, Chile. Emerg Infect Dis 2020;26: 1885-8. | Study type - single case report |
| Figueiredo LT, Souza WM, Ferres M, Enria DA. Hantaviruses and cardiopulmonary syndrome in South America. Virus Res 2014;187: 43-54. | Study type - review |
| Gauld RL, Craig JP. Epidemiological pattern of localized outbreaks of epidemic hemorrhagic fever. Am J Hyg 1954;59: 32-8. | Participants - not hantavirus |
| Glass GE. Hantaviruses. Current Opinion in Infectious Diseases 1997;10: 362-6. | Study type - review |
| Goldwasser R, Tonshoff B, Zeier M. Epidemiology of hantavirus infections in pediatric patients compared to adults in Germany in the last 10 years. Pediatric Nephrology 2011;26 (9): 1633. | Exposure - not interpersonal contact |
| Hjelle B, Spiropoulou CF, Torrez-Martinez N, Morzunov S, Peters CJ, Nichol ST. Detection of Muerto Canyon virus RNA in peripheral blood mononuclear cells from patients with hantavirus pulmonary syndrome. Journal of infectious diseases 1994;170: 1013-7. | Exposure - not interpersonal contact |
| Jaaskelainen AJ, Voutilainen L, Lehmusto R, Henttonen H, Lappalainen M, Kallio-Kokko H, et al. Serological survey in the Finnish human population implies human-to-human transmission of Ljungan virus or antigenically related viruses. Epidemiol Infect 2016;144: 1278-85. | Participants - not hantavirus |
| Kofman A, Eggers P, Kjemtrup A, Hall R, Brown S, Choi M, et al. Contract tracing investigation following first case of andes virus in the United States. Open Forum Infectious Diseases 2018;5 (Supplement 1): S761-S2. | Abstract only - not enough information to include |
| Kofman A, Eggers P, Kjemtrup A, Hall R, Brown SM, Morales-Betoulle M, et al. Notes from the Field: Contact Tracing Investigation after First Case of Andes Virus in the United States - Delaware, February 2018. MMWR Morb Mortal Wkly Rep 2018;67: 1162-3. | Study type - brief report / not enough information to include |
| Larrieu E, Cantoni G, Herrero E, Perez A, Talmon G, Vazquez G, et al. Hantavirus antibodies in rodents and human cases with pulmonary syndrome, Rio Negro, Argentina. Medicina (B Aires) 2008;68: 373-9. | Exposure - not interpersonal contact |
| Larrieu E, Herrero E, Cachau MG, Labanchi JL, Mancini S, Padula P, et al. Seroprevalencia de hantavirus en roedores y casos humanos en el sur de la Argentina. Revista Brasileira de Epidemiologia 2003;6: 68-75. | Exposure - not interpersonal contact |
| Lazaro ME, Cantoni G, Calanni L, Resa AJ, Herrero E, Iacono M, et al. TravellersIE hantavirus pulmonary syndrome in Andean Patagonia. Argentina. International Journal of Infectious Diseases 2010;14: e139. | Abstract only - not enough information to include |
| Lazaro ME, Resa AJ, Barclay CM, Calanni L, Samengo L, Martinez L, et al. [Hantavirus pulmonary syndrome in southern Argentina]. Medicina (B Aires) 2000;60: 289-301. | Exposure - not interpersonal contact |
| Lee HW. Hemorrhagic fever with renal syndrome in Korea. Rev Infect Dis 1989;11 Suppl 4: S864-76. | Study type - review |
| Leshchinskaya EV, Povalishina TP. Clinical and epidemiologic characteristics of Bolivian hemorrhagic fever (Russian). [Russian]. TrInstPolioVirusEntsef 1973;21: 118-25+213. | Study type - review |
| Limongi JE, Costa FCd, Paula MBCd, Pinto RdMC, Oliveira MdLA, Pajuaba Neto AdA, et al. Síndrome cardiopulmonar por hantavírus no Triângulo Mineiro e Alto Paranaíba, Minas Gerais, 1998-2005: aspectos clínico-epidemiológicos de 23 casos. Rev Soc Bras Med Trop 2007;40: 295-9. | Exposure - not interpersonal contact |
| MacNeil A, Ksiazek TG, Rollin PE. Hantavirus pulmonary syndrome, United States, 1993-2009. Emerg Infect Dis 2011;17: 1195-201. | Exposure - not interpersonal contact |
| Markotic A. Human-to-human transmission of hantaviruses. Lancet 1997;350: 596. | Study type - letter with no new data |
| Más M, Vázquez M, Vomero A, Pandolfo S, Dall, Orso P, et al. Síndrome pulmonar por hantavirus: primeros casos pediátricos reportados en Uruguay. Rev méd Urug 2009;25: 116-23. | Exposure - not interpersonal contact |
| McCormic ZD, Balihe MN, Havas KA, Baty SA. Puumala hantavirus outbreak among U.S. military health care beneficiaries, Stuttgart, Germany--2012. Msmr 2013;20: 12-5. | Exposure - not interpersonal contact |
| Mendes WS, da Silva AAM, Neiva RF, Costa NM, de Assis MS, Vidigal PMO, et al. Serologic survey of hantavirus infection, Brazilian Amazon. Emerging Infectious Diseases 2010;16: 889. | Exposure - not interpersonal contact |
| Mills JN, Corneli A, Young JC, Garrison LE, Khan AS, Ksiazek TG. Hantavirus pulmonary syndrome--United States: updated recommendations for risk reduction. Centers for Disease Control and Prevention. MMWR Recomm Rep 2002;51: 1-12. | Study type - recommendations / guideline to reduce risk |
| Miyamoto H, Kariwa H, Araki K, Lokugamage K, Hayasaka D, Cui BZ, et al. Serological analysis of hemorrhagic fever with renal syndrome (HFRS) patients in Far Eastern Russia and identification of the causative hantavirus genotype. Arch Virol 2003;148: 1543-56. | Exposure - not interpersonal contact |
| Monteverde MJ, Hodara K. Movimientos de roedores intra- e inter-ambiente y riesgo de exposición al Hantavirus “Andes” en Patagonia norte, Argentina. Ecología austral 2017;27: 279-89. | Participants - rats only |
| Montgomery JM, Blair PJ, Carroll DS, Mills JN, Gianella A, Iihoshi N, et al. Hantavirus pulmonary syndrome in Santa Cruz, Bolivia: outbreak investigation and antibody prevalence study. PLoS Negl Trop Dis 2012;6: e1840. | Exposure - not interpersonal contact |
| Nikolic J, Kuzman I, Markotic A, Rode OD, Curic I, Ivankovic HB, et al. The occurrence of hemorrhagic fever with renal syndrome in southern parts of Bosnia and Herzegovina. Coll Antropol 2009;33 Suppl 2: 37-42. | Exposure - not interpersonal contact |
| Nolte KB, Foucar K, Richmond JY. Hantaviral biosafety issues in the autopsy room and laboratory: concerns and recommendations. Hum Pathol 1996;27: 1253-4. | Study type - recommendations / guideline to reduce risk |
| Nunes-Araujo FR, Nishioka SD, Ferreira IB, Suzuki A, Bonito RF, Ferreira MS. Absence of interhuman transmission of hantavirus pulmonary syndrome in Minas Gerais, Brazil: evidence from a serological survey. Clin Infect Dis 1999;29: 1588-9. | Abstract only - not enough information to include |
| Orellana C. Chilean research throws light on hantavirus transmission. Lancet Infect Dis 2003;3: 8. | Study type - letter with only animal data |
| Overturf GD. Clinical sin nombre hantaviral infections in children. Pediatr Infect Dis J 2005;24: 373-4. | Study type - review |
| Padula PJ, Colavecchia SB, Martinez VP, Gonzalez Della Valle MO, Edelstein A, Miguel SD, et al. Genetic diversity, distribution, and serological features of hantavirus infection in five countries in South America. J Clin Microbiol 2000;38: 3029-35. | Exposure - not interpersonal contact |
| Pai RK, Bharadwaj M, Levy H, Overturf G, Goade D, Wortman IA, et al. Absence of infection in a neonate after possible exposure to sin nombre hantavirus in breast milk. Clin Infect Dis 1999;29: 1577-9. | Study type - single case report |
| Panos G, Sargianou M, Papa A, Gogos C. Hantaviruses: seroprevalence and risk factors among humans in Achaia prefecture, Greece. BMC infectious diseases 2014;14. | Exposure - not interpersonal contact |
| Pedrosa PB, Cardoso TA. Viral infections in workers in hospital and research laboratory settings: a comparative review of infection modes and respective biosafety aspects. Int J Infect Dis 2011;15: e366-76. | Study type - review |
| Pettersson L, Klingstrom J, Hardestam J, Lundkvist A, Ahlm C, Evander M. Hantavirus RNA in saliva from patients with hemorrhagic fever with renal syndrome. Emerg Infect Dis 2008;14: 406-11. | Exposure - not interpersonal contact |
| Pettersson L, Rasmuson J, Andersson C, Ahlm C, Evander M. Hantavirus-specific IgA in saliva and viral antigen in the parotid gland in patients with hemorrhagic fever with renal syndrome. J Med Virol 2011;83: 864-70. | Exposure - not interpersonal contact |
| Pini NC, Resa A, del Jesus Laime G, Lecot G, Ksiazek TG, Levis S, et al. Hantavirus infection in children in Argentina. Emerg Infect Dis 1998;4: 85-7. | Participants - only child contacts |
| Pizarro E, Navarrete M, Mendez C, Zaror L, Mansilla C, Tapia M, et al. Immunocytochemical and Ultrastructural Evidence Supporting That Andes Hantavirus (ANDV) Is Transmitted Person-to-Person Through the Respiratory and/or Salivary Pathways. Front Microbiol 2020;10: 2992. | Exposure - not interpersonal contact |
| Rivers MN, Alexander JL, Rohde RE, Pierce JR, Jr. Hantavirus pulmonary syndrome in Texas: 1993-2006. South Med J 2009;102: 36-41. | Exposure - not interpersonal contact |
| Schneider F, Vidal L, Auvray C, Khider Y, Graesslin O. [The first French hemorrhagic fever with renal syndrome in pregnant woman]. J Gynecol Obstet Biol Reprod (Paris) 2009;38: 440-2. | Study type - single case report |
| Silberberg L, Rollin PE, Kerouani G, Courdrier D. Haemorrhagic fever with renal syndrome and pregnancy: a case report. Trans R Soc Trop Med Hyg 1993;87: 65. | Study type - single case report |
| Silva-Vergara ML, Costa JC, Jr., Barata CH, Curi VG, Tiveron CG, Jr., Teixeira AC. Hantavirus pulmonary syndrome in Uberaba, Minas Gerais, Brazil. Mem Inst Oswaldo Cruz 2002;97: 783-7. | Exposure - not interpersonal contact |
| Sin MA, Stark K, Van Treeck U, Dieckmann H, Uphoff H, Hautmann W, et al. Risk factors for hantavirus infection in Germany, 2005. Emerging Infectious Diseases 2007;13: 1364-6. | Exposure - not interpersonal contact |
| Song G. Epidemiological progresses of hemorrhagic fever with renal syndrome in China. Chin Med J (Engl) 1999;112: 472-7. | Study type - review |
| Sosa-Estani S, Martinez VP, Gonzalez Della Valle M, Edelstein A, Miguel S, Padula PJ, et al. [Hantavirus in human and rodent population in an endemic area for hantavirus pulmonary syndrome in Argentina]. Medicina (B Aires) 2002;62: 1-8. | Exposure - not interpersonal contact |
| Tenorio Albuquerque J, Godoi M, Lemos Hinrichsen S, Tenorio Albuquerque E, Fernandes De Moura IM, Batista Juca M, et al. Leptospirosis and Hantavirus disease: Report of ten cases. [Portuguese]. Revista Brasileira de Medicina 2005;62: 450-3. | Exposure - not interpersonal contact |
| Tercas ACP, dos Santos MA, Pignatti MG, Espinosa MM, de Melo Via AVG, Menegatti JA. Hantavirus pulmonary syndrome outbreak, Brazil, December 2009- January 2010. Emerging Infectious Diseases 2013;19: 1824-7. | Exposure - not interpersonal contact |
| Tsai TF. Hemorrhagic fever with renal syndrome: mode of transmission to humans. Lab Anim Sci 1987;37: 428-30. | Study type - review |
| Umenai T, Lee HW, Lee PW, Saito T, Toyoda T, Hongo M, et al. Korean haemorrhagic fever in staff in an animal laboratory. Lancet 1979;1: 1314-6. | Exposure - not interpersonal contact |
| Vapalahti K, Paunio M, Brummer-Korvenkontio M, Vaheri A, Vapalahti O. Puumala virus infections in Finland: increased occupational risk for farmers. Am J Epidemiol 1999;149: 1142-51. | Exposure - not interpersonal contact |
| Vapalahti K, Virtala AM, Vaheri A, Vapalahti O. Case-control study on Puumala virus infection: smoking is a risk factor. Epidemiol Infect 2010;138: 576-84. | Exposure - not interpersonal contact |
| Vial PA, Valdivieso F, Mertz G, Castillo C, Belmar E, Delgado I, et al. Incubation period of hantavirus cardiopulmonary syndrome. Emerg Infect Dis 2006;12: 1271-3. | Exposure - not interpersonal contact |
| Wang X, Shen W, Qin Y, Ying L, Li H, Lu J, et al. A case-control study on the risk factors for hemorrhagic fever with renal syndrome. BMC Infect Dis 2020;20: 103. | Exposure - not interpersonal contact |
| Watson DC, Sargianou M, Papa A, Chra P, Starakis I, Panos G. Epidemiology of Hantavirus infections in humans: a comprehensive, global overview. Crit Rev Microbiol 2014;40: 261-72. | Study type - review |
| Weber DJ, Rutala WA. Risks and prevention of nosocomial transmission of rare zoonotic diseases. Clin Infect Dis 2001;32: 446-56. | Study type - review |
| Webster D, Lee B, Joffe A, Sligl W, Dick D, Grolla A, et al. Cluster of cases of hantavirus pulmonary syndrome in Alberta, Canada. Am J Trop Med Hyg 2007;77: 914-8. | Exposure - not interpersonal contact |
| Xu ZY, Tang YW, Kan LY, Tsai TF. Cats--source of protection or infection? A case-control study of hemorrhagic fever with renal syndrome. Am J Epidemiol 1987;126: 942-8. | Exposure - not interpersonal contact |
| Young JC, Mills JN, Enria DA, Dolan NE, Khan AS, Ksiazek TG. New World hantaviruses. Br Med Bull 1998;54: 659-73. | Study type - review |
| Zambrano A, Peralta R, Carlaga M. [Case Report: first case of hantavirus cardiopulmonary syndrome diagnosed in the Second Region of Antofagasta]. Rev Chilena Infectol 2012;29: 477. | Study type - single case report |
| Zeitz PS, Butler JC, Cheek JE, Samuel MC, Childs JE, Shands LA, et al. A case-control study of hantavirus pulmonary syndrome during an outbreak in the southwestern United States. J Infect Dis 1995;171: 864-70. | Exposure - not interpersonal contact |
| Zhang Y, Dong X, Yuan J, Zhang H, Yang X, Zhou P, et al. Hantavirus outbreak associated with laboratory rats in Yunnan, China. Infection, Genetics and Evolution 2010;10: 638-44. | Exposure - not interpersonal contact |

**No full text could be found (N=14)**

1. Cascón A, Leoni C. El síndrome pulmonar por hantavirus (SPH) como enfermedad profesional. Salud ocup (Buenos Aires) 1999;17: 4-15.
2. Chen HL, Yang JY, Chen HY, Lin TH, Wang GR, Horng CB. Surveillance of anti-hantavirus antibodies among certain high-risk groups in Taiwan. Journal of the Formosan Medical Association 1998;97: 69-72.
3. Diglisic G, Rossi CA, Doti A, Walshe DK. Seroprevalence study of Hantavirus infection in the community based population. Md Med J 1999;48: 303-6.
4. Kimmig P, Silva-Gonzalez R, Backe H, Brockmann S, Oehme R, Ernst E, et al. [Epidemiology of hantaviruses in Baden-Wurttemberg]. Gesundheitswesen 2001;63: 107-12.
5. Knobloch J, Dietrich M, Peters D, Nielsen G, Schumacher HH. [Maridi haemorrhgic fever: a new viral disease (author's transl)]. Dtsch Med Wochenschr 1977;102: 1575-81.
6. Liu YF, Yan PS, Wang BY, Liu J, Wang NP, Zhu XS, et al. Intrauterine infection of epidemic hemorrhagic fever (EHF) via placenta. Chin Med J (Engl) 1987;100: 756-8.
7. Macías G. Infección por hantavirus en Buenos Aires, Argentina. Prensa méd argent 2005;92: 127-33.
8. Mesic S, Almedin H. [Investigation of modes of hantavirus infection transmission from rodents to humans]. Med Arh 2008;62: 229-30.
9. Moolenaar RL, Breiman RF, Peters CJ. Hantavirus pulmonary syndrome. Semin Respir Infect 1997;12: 31-9.
10. Mutnykh ES, Dzagurova TK, Bernshtein AD, Kalinkina EV, Korotina NA, Apekina NS, et al. [The epidemiological, epizootological, and etiological characteristics of the 2006-2007 outbreak of hemorrhagic fever with renal syndrome in the Tambov Region]. Vopr Virusol 2011;56: 43-7.
11. Nurgaleeva RG, Tkachenko EA, Stepanenko AG, Mustafin IM, Kireev SG, Dzagurova TK, et al. [An epidemiological analysis of hemorrhagic fever with renal syndrome morbidity in the Republic of Bashkortostan in 1997]. Zh Mikrobiol Epidemiol Immunobiol 1999: 45-9.
12. Padula PJ, Edelstein A, Miguel SD, Lopez NM, Rossi CM, Rabinovich RD. [Epidemic outbreak of Hantavirus pulmonary syndrome in Argentina. Molecular evidence of person to person transmission of Andes virus]. Medicina (B Aires) 1998;58 Suppl 1: 27-36.
13. Peng H, Tang S, Qi X. [Clinical study on intrauterine hemorrhagic fever with renal syndrome virus infection]. Zhonghua Shi Yan He Lin Chuang Bing Du Xue Za Zhi 2002;16: 281-2.
14. Wang RF. [Studies on some epidemiological factors of epidemic hemorrhagic fever (by means of matched case-control studies and sequential analysis (author's transl)]. Zhonghua Yu Fang Yi Xue Za Zhi 1980;14: 228-30.

**Duplicates not picked up at previous stage (N=6)**

1. Castillo H C, Mardones M J, Villagra C E. Prevalence of antibodies to hantavirus in health care workers exposed to patients with hantavirus pulmonary syndrome in Temuco, Chile. Revista médica de Chile 2000;128: 735-9.
2. Limongi JE, da Costa FC, de Paula MB, Pinto Rde M, Oliveira Mde L, Pajuaba Neto Ade A, et al. [Hantavirus cardiopulmonary syndrome in the Triangulo Mineiro and Alto Paranaiba regions, State of Minas Gerais, 1998-2005: clinical-epidemiological aspects of 23 cases]. Rev Soc Bras Med Trop 2007;40: 295-9.
3. Limongi JE, Da Costa FC, De Paula MBC, Pinto RDMC, Oliveira MDLA, Neto ADAP, et al. Hantavirus cardiopulmonary syndrome in the Triangulo Mineiro and Alto Paranaiba regions, State of Minas Gerais, 1998-2005: Clinical-epidemiological aspects of 23 cases. [Portuguese]. Revista da Sociedade Brasileira de Medicina Tropical 2007;40: 295-9.
4. Macias G, Gulotta H, San Juan J, Videla J, Santucho E, Troncoso A. Hantavirus infection in Buenos Aires, Argentina. [Spanish]. Prensa Medica Argentina 2005;92: 127-33.
5. Padula PJ, Edelstein A, Miguel SDL, Lopez NM, Rossi CM, Rabinovich RD. Brote epidemico del sindrome pulmonar por Hantavirus en la Argentina: evidencia molecular de la transmission persona a persona del virus Andes. Medicina (BAires) 1998;58: 27-36.
6. Pizarro E, Navarrete M, Mendez C, Zaror L, Mansilla C, Tapia M, et al. Immunocytochemical and Ultrastructural Evidence Supporting That Andes Hantavirus (ANDV) Is Transmitted Person-to-Person Through the Respiratory and/or Salivary Pathways. Frontiers in Microbiology 2020;10 (no pagination).

# **Supplementary File 3:** Risk of bias assessment

## **Risk of bias assessment - part A**

| **Study** |  | **Bayard 2004A** |  | **Bayard 2004B** |  | **Chaparro 1998** |
| --- | --- | --- | --- | --- | --- | --- |
| **Domains** | **RoB** | **Rationale** | **RoB** | **Rationale** | **RoB** | **Rationale** |
| Confounding | Crit | Cross-sectional study, no multivariate analysis to control for potential confounders, e.g. rodent exposure, duration or type or time (i.e. disease stage) of contact. | Crit | Cross-sectional study, no multivariate analysis to control for potential confounders, e.g. use of PPE, duration or type or time (i.e. disease stage) of contact. | Crit | Cross-sectional study, no multivariate analysis to control for potential confounders, e.g. use of PPE, duration or type or time (i.e. disease stage) of contact. |
| Selection | Ser | Selection into the study was based on knowledge of exposure and possible knowledge of outcome. No adjustment was made for this in the analysis. | Ser | Selection into the study was based on knowledge of exposure and no response rate (denominator) is reported. | Low | Selection into the study was independent of exposure because all hospital staff were invited to participate. The response rate was high (87.9%) though there was some variation between exposure areas, ranging from 71.4% among laboratory staff to 89.7% of other administrative staff. |
| Measurement of exposures | Crit | Exposure was measured retrospectively by interview and could have been affected by knowledge of risk of the outcome. No clear definition of exposure. Analysis was based on living in the same household or not, but some neighborhood participants were also exposed to the index patient. | Crit | Exposure was measured retrospectively by self-report questionnaire and could have been affected by knowledge of risk of the outcome. | Crit | Exposure was measured retrospectively by self-report questionnaire and could have been affected by knowledge of risk of the outcome. |
| Departures from exposures | Ser | Potential for differential misclassification due to limited information about duration, type or time of contact and co-exposure to infected rodents. This was not adjusted for in the analysis. | Ser | Potential for differential misclassification due to limited information about duration, type or time of contact, co-exposure to rodents and use of PPE. This was not adjusted for in the analysis. | Ser | Potential for differential misclassification due to limited information about duration, type or time of contact, co-exposure to rodents and use of PPE. This was not adjusted for in the analysis. |
| Missing data | Mod | Not clear who was missing data. Authors state that both interview and serum specimens were obtained from 83% of the residents. | Ser | Not clear who was missing data and no response rate was reported. | Mod | Not clear who was missing data. Authors state that both interview and serum specimens were obtained from 88% of the hospital staff. |
| Measurement of outcomes | Mod | No mention of blinding of outcome assessment. Only IgG-positive survey participants were also tested for IgM antibodies thus allowing for under detection of more recent exposure. | Mod | No mention of blinding of outcome assessment. Only IgG-positive survey participants were also tested for IgM antibodies thus allowing for under detection of more recent exposure. | Low | No mention of blinding of outcome assessment. |
| Reported results | Mod | IgM and IgG positive/negative are standard outcomes for this type of study, but the selection of exposure groups (household vs neighbourhood) doesn't consider the inclusion of neighbourhood participants who also had contact with the index case (n=6). | Low | IgM and IgG positive/negative are standard outcomes for this type of study. | Low | IgM and IgG positive/negative are standard outcomes for this type of study. |
| **Overall bias** |  | **Critical** |  | **Critical** |  | **Critical** |

Crit – critical; Mod – moderate; RoB – risk of bias assessment; Ser – serious;

## **Risk of bias assessment - part B**

| **Study** |  | **Ferres 2007** |  | **Pini 2003** |  | **Ruo 1994** |
| --- | --- | --- | --- | --- | --- | --- |
| **Domains** | **RoB** | **Rationale** | **RoB** | **Rationale** | **RoB** | **Rationale** |
| Confounding | Ser | No randomization. However, the study was a prospective cohort and a multivariate analysis was conducted that considered all potential confounders. | Crit | Cross-sectional study, no multivariate analysis to control for potential confounders, e.g., rodent exposure, duration or type or time (i.e., disease stage) of contact. | Ser | No randomization. However, the study included both a cross-sectional sample and a prospective cohort and a multivariate analysis was conducted that considered all potential confounders. |
| Selection | Mod | All participants were selected based on being a contact of the index case and before the disease status was known. However, the response rate was not reported, and it is not reported if some contacts declined to participate. | Mod | Selection into the study was independent of exposure because a selected sample of the population was invited to participate. However, no response rate was reported. | Mod | Selection into the study was independent of exposure because a selected sample of the population was invited to participate. However, the response rate was low - 64·7% in the cross-sectional study and 83·2% for the cohort study. |
| Measurement of exposures | Mod | Exposure was measured by an administered questionnaire prior to knowledge of the outcome. Detailed information was collected about the type of contact. | Crit | Exposure was measured retrospectively by self-report questionnaire and could have been affected by knowledge of risk of the outcome. | Ser | Exposure was measured retrospectively by self-report interview in the cross-sectional study and then again in the follow-up of the cohort. Responses could have been affected by knowledge of risk of the outcome. |
| Departures from exposures | Mod | While there might have been some misclassification it is not expected to be differential or impact significantly on the results. Co-exposures and confounders were adjusted for in the analyses. | Ser | Potential for differential misclassification due to limited information about duration, type or time of contact and co-exposure to infected rodents. This was not adjusted for in the analysis. | Ser | Potential for differential misclassification due to limited information about duration, type or time of contact and co-exposure to infected rodents. Co-exposure to a HFRS patient was not adjusted for in the analysis. |
| Missing data | Mod | Not clear if there was any missing exposure data. No statement was made. | Mod | Not clear if there was any missing exposure data. No statement was made. | Ser | Not reported clearly as to who was missing data and how many could be included in the multivariate analysis. There was clearly missing data in the univariate analysis, which could have been more substantial in the multivariate analysis. |
| Measurement of outcomes | Low | No mention of blinding of outcome assessment. | Low | No mention of blinding of outcome assessment. | Low | No mention of blinding of outcome assessment. |
| Reported results | Low | IgM/IgG and RT-PCR positive/negative are standard outcomes for this type of study. | Low | IgM and IgG positive/negative are standard outcomes for this type of study. | Low | IgM and IgG positive/negative are standard outcomes for this type of study. |
| **Overall bias** |  | **Serious** |  | **Critical** |  | **Serious** |

Crit – critical; Mod – moderate; RoB – risk of bias assessment; Ser – serious;

## **Risk of bias assessment - part C**

| **Study** |  | **Vitek 1996** |  | **Williams 1997** |
| --- | --- | --- | --- | --- |
| **Domains** | **RoB** | **Rationale** | **RoB** | **Rationale** |
| Confounding | Crit | Cross-sectional study, no multivariate analysis to control for potential confounders, e.g., use of PPE, duration or type or time (i.e., disease stage) of contact. | Crit | Cross-sectional study, no multivariate analysis to control for potential confounders, e.g., rodent exposure, duration or type or time (i.e. disease stage) of contact. |
| Selection | Mod | Selection into the study was likely biased towards those with known exposure and the response rate varied between exposure areas, ranging from 70% among laboratory workers to 97% for those performing autopsies). | Crit | Selection into the study was nonrandom and by convenience. Subjects with known history of an HPS-type illness were excluded. Only the available family and household contacts of 3 case-patients were enrolled. No adjustment was made for this in the analysis. |
| Measurement of exposures | Crit | Exposure was measured retrospectively by self-report questionnaire and could have been affected by knowledge of risk of the outcome. | Crit | Exposure was based on being a current family or household member of only 3 of the case-patients. |
| Departures from exposures | Ser | Potential for differential misclassification due to limited information about duration, type or time of contact and use of PPE. This was not adjusted for in the analysis. | Ser | Potential for differential misclassification due to limited information about duration, type or time of contact and co-exposure to infected rodents. This was not adjusted for in the analysis. |
| Missing data | Low | Exposure information was missing for 5% of those with serum specimens. | Mod | Not clear if there was any missing exposure data. No statement was made. |
| Measurement of outcomes | Mod | No mention of blinding of outcome assessment. No details provided on the sensitivity of the ELISA test but it could be low given that no positives were found. | Low | No mention of blinding of outcome assessment. |
| Reported results | Low | IgM and IgG positive/negative are standard outcomes for this type of study. | Low | IgM and IgG positive/negative are standard outcomes for this type of study. |
| **Overall bias** |  | **Critical** |  | **Critical** |

Crit – critical; Mod – moderate; RoB – risk of bias assessment; Ser – serious;

# **Supplementary File 4:** Description of HPS cases, Rio Negro Province, Argentina 1996

**HPS Cases, Rio Negro Province, Argentina 1996**

Between September 22 and December 5, 1996, 18 cases of HPS occurred in residents of, or visitors to, the towns of El Bolsón, Bariloche, and Esquel in the Rio Negro Province of Argentina, which is in the southwest of the country in the Patagonia Region. Two additional persons who had contact with El Bolsón patients but had not visited the area, contracted HPS during this period [1]. Results of analyses of cluster/s of cases within this outbreak have been presented in four different papers [1-4]. These papers are often cited in the hantavirus literature by these authors, and other authors, as proof of human-to-human transmission of HPS.

Given that these four papers are all analyses of the same 20 cases (or subgroups of these 20 cases), they need to be considered as one study rather than as four separate studies. The table below is a summary of information provided by the four papers for each of the 20 cases. Inconsistencies between the reports are also noted. For the systematic review, the 1997 paper by Wells et al. [1] is considered to be the primary reference because it includes all 20 cases and is the earliest complete report of the outbreak. The other three papers are considered secondary references [2-4]. A fifth related paper reports a serological survey of healthcare workers and other residents of El Bolsón and Bariloche that is associated with the outbreak and was conducted in 1996 [5]. This paper is reported as a separate study because it had a different design and included different participants.

***Summary of findings:***

Sixteen cases (I, A, B, P, L, Q, K, J, H, F, O, N, C, M, E, D), designated Epilink/96, had the same sequence in the M segment G1a, G1b, G2, and in 39 NCR S fragment [4]. The two El Bolsón human case sequences, G and T, belonging to contemporary but non-epidemiologically related patients, differed from Epilink/96. The detection of the same sequence in different people might be explained by infection with a genetically identical hantavirus. The more geographically distant the cases, the more unlikely it is that a unique source of infection or same variant within the rodent local populations occurred. Following this reasoning, infection could have occurred through human-to-human transmission. Of the 16 cases, the authors claim that 2 are likely to be from human-to-human transmission (Doctor D and Case M). Doctor D had never been to the area. Case M had never visited El Bolsón but traveled by car for 20 h with a symptomatic infected person (L) and stayed with her infected parents (J and H). No evidence of rodent infestation was found when the car was examined 3 weeks later. No other HPS cases were detected in Buenos Aires or Bariloche during that time. Finding the same sequence in the patients and their contacts in the hospital and the lack of other HPS cases in these cities were regarded by the authors as strongly suggesting human-to-human transmission of the virus [4]. Four cases exposed to cases with HPS in Bariloche hospital (N, O, P, and Q) may be from human-to-human transmission as no other cases of HPS were found in Bariloche [4]. However, co-exposure to rodents cannot be ruled out.

**HPS Cases Compared in the 1996 outbreak in Rio Negro Province, Argentina**

| **Case E1996** | **Case C1997** | **Case W1997** | **Case P1998** | **Residence** | **Relationship and type of contact with previous cases up to 45 days before onset** | **Rodent exposure** | **Date of onset^p^** | **Days symptom onset/contact^w^** |
| --- | --- | --- | --- | --- | --- | --- | --- | --- |
| N=5 | N=12 | N=20 | N=16 |  |  |  |  |  |
|  | C1 | I | I | El Bolsón^a^ | NKC. Male^c^ | Shared risky work with B and L near home of I^c^  Shared an activity with K in a high-risk area far from the home of I^c^ | 9-22-96 † |  |
| A | C2 | A | A | El Bolsón^a^ | Doctor of I. |  | 10-12-96 † | 20 |
| B | C3 | B | B | El Bolsón^a^ | I’s mother. 70 years old^w^ | Shared risky work with I and L near home of I^c^ | 10-13-96 † | 21 |
|  |  | P | P | Bariloche^a^ | Visiting Bariloche Private Hospital at the same time El Bolson cases were hospitalized. |  | 10-13-96 † |  |
|  | C4 | L | L | El Bolsón^a^ | Housekeeper of I and B. Travelled with J and H in a car.^c^ | Shared risky work with I and B near home of I^c^ | 10-21-96 † |  |
|  |  | Q | Q | Bariloche^a^ | Spouse of P. Staying at Bariloche Private Hospital giving birth when El Bolson cases were being transferred. |  | 10-22-96 † |  |
|  | C5 | K | K | El Bolsón | I’s friend. Visited him when hospitalized. | Shared an activity with I in a high-risk area far from the home of I^c^ | 10-23-96 |  |
|  | C8 | J | J | Buenos Aires^a,b^ | I’s brother-in-law. Stayed at B’s home during her funeral. Traveled with L and H in a car. |  | 10-31-96 | 11 |
|  | C9 | H | H | Buenos Aires^a,b^ | I’s sister, spouse of J. Stayed at B’s home during her funeral. Traveled with L and J in a car. |  | 11-4-96 | 15 |
|  | C6 | F | F | El Bolsón | Doctor of I, B, and G; daily contact with A. He intubated patient B and examined patients I and G. He also had daily contact with his colleague, (patient A), and spoke briefly with patient I’s sister (patient H), brother-in-law (patient J), and friend (patient K).^w^ |  | 11-7-96 |  |
|  |  | O | O | Bariloche | Visiting a non-HPS case patient at Bariloche Private Hospital. |  | 11-7-96 † |  |
|  |  | N | N | Bariloche | Bariloche Private Hospital receptionist when El Bolsón cases were hospitalized. Friendly relation with P and Q |  | 11-8-96 † | 17 (after Q)  26 (after P) |
| C | C7 | C | C | El Bolsón^a^ | Spouse of A, transferred to Buenos Aires Hospital. |  | 11-8-96 | 27 |
|  | C10 | M | M | Buenos Aires^a^ | J’s and H’s daughter. Traveled by car with L, J, and H (No history of visiting the area). | No evidence of rodent infestation was found when the car was examined 3 weeks later… patient M had not been to El Bolsón in the preceding months, traveling only as far as Jacobacci (~250 miles from El Bolsón) for patient B’s funeral.^w^ | 11-28-96 | 29 (after J) |
| E | C11 | E | E | Buenos Aires^b^ | Friend of C, also a doctor. Took care of her daily at Buenos Aires Hospital. 40 years old.^w^ | She spent 3 days in El Bolsón after the death of patient A.^w^ | 11-28-96 † | 17 |
| D | C12 | D | D | Buenos Aires | Doctor of C at Buenos Aires Hospital. | The doctor had not traveled outside Buenos Aires, and she reported no contact with rodents during the 2 months preceding her illness. | 12-5-96 | 24 |
|  |  | G |  | El Bolsón | NKC. 44 years old.^w^ |  | 9-23-96 |  |
|  |  | T |  | El Bolsón | NKC. 14 years old.^w^ |  | 10-4-96 |  |
|  |  | U |  | Esquel | NKC. 33 years old.^w^ | Worked in Chubut Province (150km south of El Bolsón) | 11-20-96 † |  |
|  |  | R |  |  | 29 years old.^w^ | Lived in the Chilean mountains and had prodromal symptoms of HPS on arrival in El Bolsón.^w^ |  |  |

Table adapted from [4] (P1998)^p^, with additional information and case numbers added from [3] (E1996)^e^, [2] (C1997)^c^, and [1] (W1997)^w^.

Note. NKC, no known contacts with HPS cases.

^a^ Living with another HPS case. Housemate case groups: (I, his mother B, and their housekeeper L); (P and his spouse Q), (A and his spouse C); (M and their parents J and H).

^b^ Visitors to El Bolsón.

† Died from HPS

**Discrepancies between reports.**

Enria 1996 reports that doctor B survived, while Padula 1998 and Wells 1997 report that she died.

Doctor D – description of contact with C

- Enria 1996 – “In the emergency unit of a hospital in Buenos Aires, during the process of arterial bleeding from C, doctor D suffered accidental exposition to the blood of the patient, without cuts or wounds. Blood from patient C entered into contact with the hands-without gloves-of doctor D.”
- Wells 1997 – “…an admitting doctor (patient D) spent 1 hour taking a clinical history and examining her. The doctor (patient D) applied pressure to a venipuncture site on patient C’s arm with multiple layers of gauze; no obvious blood contact occurred. The only other contact between this doctor and patient C occurred 2 days later, when the doctor briefly visited the hospital’s intensive care unit to attend to another patient.”

Doctor E – description of contact and role with C

- Enria 1996 – “Doctor E, who died, had been friend of C and presented two known risk factors. The first risk factor was a visit to El Bolsón performed during the burial of A, husband of C, 50 days previous to her admission with HPS; the second risk factor was looking after C three to four hours daily

during her hospitalization in Buenos Aires.”

- Wells 1997 – “She spent 3 days in in El Bolsón after the death of patient A. She visited patient C often in the hospital but was not directly involved

in the clinical management of any HPS patients.”

- Cantoni 1996 – “a physician who went to El Bolsón to attend case 7.” Note: case 7 is patient C.

Enria 1996 – reports that time between first contact with C and developing symptoms was 27 days for D and 28 days for E. Wells 1997 reports 24 days for D and 17 days for E after C was admitted to hospital in Buenos Aires. The difference for E maybe due to the time that C and E spent together in El Bolsón.


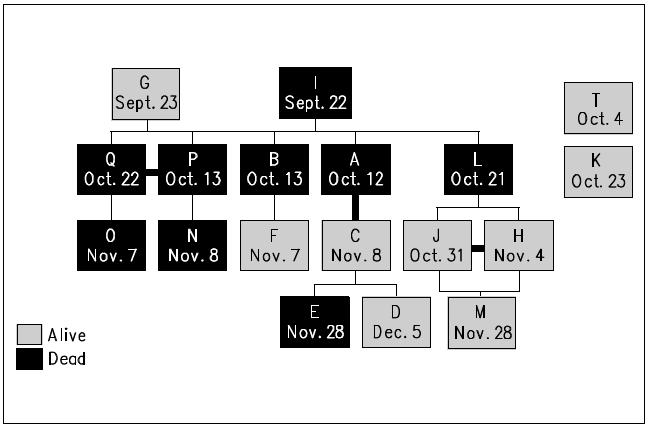


Enria 1996

Cantoni 1997

Wells 1997 & Padula 1998

**Figure 1**. Transmission tree for HPS cases in southern Argentina, September–December 1996, indicating dates of onset of symptoms, survivor status, and proposed lines of transmission. Lines of transmission are hypothetical since many of the patients had contact with multiple HPS patients. Bold lines denote husband and wife. The two sporadic cases, U and R, are not shown.

**Source:** Wells et al 1997 [1] with additional information from Cantoni et al. 1997, Enria et al. 1996 and Padula et al. 1998 [2-4].

# **Supplementary Table 1:** Characteristics of included studies – non-comparative designs by country of study

| **Study ID^a,b^ reference, country** | **Region within country & year of study** | **Study type description** | **Total N (N index, N exp.)** | **Participants describe** | **Exposure definition & actual** |
| --- | --- | --- | --- | --- | --- |
| Argentina – Andes virus | |  |  |  |  |
| Alonso 2020 [6] | Rio Negro  2014 | Epidemiological investigation of 3 clustered cases. | N=3 (1, 2) | Cluster - P1/P2 were twin brothers (71 years), P3 was a female nurse of P2 (53 years). | ND. Actual: For P1/P2 - twin brothers who lived in the same house where they shared the same room and bed; both wood collectors in a forest area. P3 was a nurse who attended P2 during his initial hospitalization at a primary healthcare center. |
| Iglesias 2016 [7] | Buenos Aires province  2009-2014 | Epidemiological investigation of incidence and distribution and further investigation of 3 clusters, including possible risk activities. | N=88 (3, 3) | 88 confirmed cases. Age range 1-72 years. Of the 88 confirmed cases, 3 clusters of 2 cases each were identified, with a marital relationship between the two. The 3 index cases were male, and the contacts were female. | ND. Actual: marital relationship. 2 couples lived in the same house (clusters A1 and A2) and 1 couple (A3) had different houses in another province but both of them reported maintaining contact at the time of the hospitalization of the index case. |
| Lazaro 2007 [8] | Rio Negro, Neuquen, Chubut, and Santa Cruz provinces  1993-2005 (not 1996) | Epidemiological investigation of 9 clusters using registers of case reports, environmental and epidemiological case investigations, and clinical data. | N=51 (8, 10) | 51 cases - 49 confirmed. 20 (of 51) cases were grouped into 9 clusters; age range 1-63 years, 44∙5% male; 1 type A cluster (n=2) – infections <1-week b/n cases; 8 type B clusters (n=18) – infections >2 weeks b/n cases. | ND. Actual: association of a patient with a confirmed HPS (index case-patient) within 6 weeks of the onset of symptoms. |
| Martinez 2005 [9-11] | Buenos Aires province, Central Region and Patagonia region  2002 | Epidemiological investigation of 4 clusters of 13 cases, including genetic characterization. | N=13 (4, 9) | From July to December 2002, 31 HPS cases were reported in Argentina. 13 (of 31) were included in this study because they occurred as linked cases grouped in 4 clusters.^c^ Age range 11-58 years, 77% male. | ND. Actual: living in same house, spending time in same house, shared 14-hour bus trip and taxi ride, visit of friend to home and hospital. |
| Martinez 2020 [12] | Chubut Province  2018-2019 | Epidemiological investigation of 34 linked cases, including genetic characterization. | N=34 (10, 33?) | 34 confirmed cases. Age range 2-90 years, 44% male. | ND. Actual: contact with a HPS case, either through attending the same birthday party, wake, or hospital. |
| Wells Arg 1997 [1-4] | Rio Negro Province, Central Argentina and Buenos Aires city  1996 | Epidemiological investigation of an outbreak (Sept-Dec 1996): analysis of clinical records, interviews with survivors and/or relatives of patients, and rodent trapping. 16 (of 20) cases were epidemiologically linked (clustered). | N=16 (1, 15) | 16 (of 20) cases associated with the outbreak. Age range 13-70 years, 50% male. | ND. Actual: contact with a HPS case, either through sharing a house, marital contact, healthcare contact with HPS cases, or sharing a car trip. |
| Wells 1998 [5] | Rio Negro, 1996 | Serological survey of HCW and other residents following an outbreak. | N=524 (16, 524) | 524 people including: (1) 150 HCWs from the hospitals in El Bolsón and Bariloche; (2) 294 residents of El Bolsón representing 164 randomly selected households, all ages, 44% male; (3) 67 residents of ‘‘control’’ households from El Bolsón and Bariloche who participated in a case-control study as part of the outbreak investigation; and (4) 13 asymptomatic household contacts of confirmed HPS patients. | ND. Actual: Reported having had contact with an HPS patient (random population sample only). |
| Chile – Andes virus | |  |  |  |  |
| Castillo 2004 [13, 14] | Temuco  1998-2000 | Cross-sectional seroprevalence and epidemiologic study among family contacts and healthcare worker contacts of index cases. | N=215 (20, 215) | N=106 family members, mean age 31∙0 years, 65∙7% males, 76∙2% lived in rural areas.  N=109 HCWs, mean age 39∙3 years, 32% males, only 1 (1%) lived in rural areas. | Family member – anyone who shared housing or lodging for ≥ 1 day or stayed in closed facilities for > 1 hour with an HPS patient during the prodromal or cardiovascular stage of the disease.  HCW – caring directly for a patient, performing intubation, suctioning of respiratory secretions, cardiopulmonary resuscitation, and venous or arterial punctures, handling body fluids (blood, urine, respiratory secretions), or performing an autopsy. |
| Martinez 2014 [15] | Los Rios Region, Central Chile  2011 | Epidemiological investigation of a cluster of 5 cases, including 2 persons involved in healthcare. Clinical history and epidemiologic questionnaires; rodent trapping at 2 sites of exposure. | N=5 (1, 4) | A - 73 years, male; B - 31 years, female, nursing assistant at hospital 1; C - 53 years, female, wife of A; D - 60 years, female, housekeeper at hospital 1; E - 34 years, male, husband of B. | ND. Actual: spouse or cared for A during his prodromal phase in hospital 1. |
| Toro 1998 [16] | Aysen Region, Central Chile  1997-1998 | Epidemiological investigation of 3 family clusters, including review of clinical charts, interviews with family members and rodent trapping for 2 clusters. Serological testing of 53 contacts of 14 case patients. | N=25 (3, 8) | 25 HPS patients including three family clusters, identified in Chile b/n 1 July 1997 and 22 Jan 1998. Cluster 1 included 5 members - a father (39 years), mother, 2 sons (2 and 12 years) and a brother-in-law. Cluster 2 included 4 members of a household (no further information). Cluster 3 included a husband and wife. | ND. Actual: living in same house |
| Germany - PUUV and DOBV | |  |  |  |  |
| Hofmann 2012 [17] | 2007 & 2009 | Investigation of 4 cases of pregnant women with hantavirus and possibility of vertical transmission to their newborns. | N=8 (4, 4) | 4 pregnant women between 14 and 28 weeks of gestation: 2 infected with DOBV, ages 38 and 23; and 2 infected with PUUV, ages 40 and 33; and their 4 newborns. | Vertical transmission - mother to fetus/newborn |
| South Korea – Hantaan virus | | |  |  |  |
| Park 2019 [18] | Seoul  2017 | Epidemiological investigation of 6 HCWs with 1 or more symptoms corresponding to a febrile illness exposed to 1 patient with HFRS. | N=7 (1, 6) | The index patient was a 55-year-old man who lived in Seoul. He was admitted with high fever (39∙7°C) on November 13, 2017 and died November 19, 2017. 6 HCWs who had cared for the patient (exposed) with 1 or more symptoms corresponding to a febrile illness. | ND. Actual: HCWs who had cared for the patient and had 1 or more symptoms corresponding to a febrile illness (headache, fever and/or myalgia) were included. Exposed HCWs who did not display symptoms were excluded. |
| United States of America - Sin Nombre virus | | |  |  |  |
| Howard 1999 [19] | 1993-1998 | Investigation of 5 cases of pregnant women with hantavirus and possibility of vertical transmission to their newborns. | N=10 (5, 5) | 5 pregnant women between 13 and 29 weeks of gestation - 2 were American Indian, 2 were non-Hispanic white, and 1 was Hispanic. Age range 20-34 years, previously healthy. | Vertical transmission - mother to fetus/newborn. |
| Wells USA 1997 [20] | 1987-1995 | Epidemiological investigation of 5 clusters using registers of case reports, clinical data, environmental and epidemiological case investigations. | N=12 (5, 7) | 12 participants included in 5 clusters; 9 males; 11 were adults aged 19-57 years, 1 child aged 4 years. The association with index cases was due to work or living arrangements/familiar. | ND. Actual: worked together, lived together, or lived at separate times in the same trailer home. |

exp. – exposed; HCW – healthcare workers; ND – not defined; RT-PCR - reverse-transcription polymerase chain reaction.

^a^ If a study had more than one reference, we awarded one reference the status of primary reference. The Study ID was taken from the first author of the primary reference and year of publication.

^b^ No conflicts of interest were apparent in any of the studies.

^c^ These 4 clusters are not included in the Lazaro 2007 study that included the year 2002.

# **Supplementary Table 2:** Results – non-comparative designs by country of study

| **Study ID^a^ reference, country** | | **Outcomes measured** | | **Confounders measured** | **Description of results** | |
| --- | --- | --- | --- | --- | --- | --- |
| Argentina – Andes virus | | | |  | |  |
| Alonso 2020 [6] | | Genetic sequencing | | None | H-to-H transmission for P2/P3 appeared likely, though possible co-exposure to rodents not measured. Comparative analysis showed 100% nucleotide identity in the whole genome between the samples from patients P2 and P3 (P2/P3 genome). Patient P1 had 100% nucleotide identity in the complete S and M segments with P2/P3 but had 2 nucleotide changes in the L segment (99∙95% nucleotide identity); both differences were silent mutations. | |
| Iglesias 2016 [7] | | Serology – IgM and IgG, Virology – RT-PCR,  Genetic sequencing | | co-exposure to infected rodents | Eighty five of 88 (97%) confirmed cases showed no evidence of H-to-H transmission. Of 88 confirmed cases, 3 clusters (3∙4%) of 2 persons each were identified (A1, A2, A3). H-to-H transmission appeared likely for clusters A1 and A2 due to the infection in the index case likely occurring at the summer holiday area (A1) or workplace (A2) and time between infections. Not clear for A3 due to possible exposure to rodents. The period of onset of the disease between the index (A1, A2, A3) and contact cases was 24, 23, and 27 days for A1, A2 and A3, respectively. From the analysis of the partial nucleotide sequences of the viral genome the AND-BsAs genotype was identified and 100% identity was verified within each cluster. | |
| Lazaro 2007 [8] | | Serology - IgM and IgG, Virology – RT-PCR | | co-exposure to infected rodents | One type A cluster of two boys who shared common risk on holiday. 8 type B clusters, including 10 contacts and 8 index cases - range 19-40 days between symptom onset. The authors conclude that airborne transmission could not be excluded for any cluster. The authors claim that in 6 of the 8 type B clusters that H-to-H transmission was probable due to the time between infections and no evidence for rodent exposure by contacts was found (B1, B2 for 1/3 contacts, B4, B5, B7 and B8). None of the trapped rodents were seropositive for hantavirus. | |
| Martinez 2005 [9-11] | | Serology - IgM and IgG, Virology – RT-PCR,  Genetic sequencing | | co-exposure to infected rodents | H-to-H transmission appeared likely for C1-s and C4-c from clusters C1 and C4; but not for C2 and C3. Clusters 2 and 3 clearly had a common exposure to rodents and all members of the cluster became ill within 12 or 19 days of each other. Cluster 1 (C1) was a father-son pair (C1-f and C1-s) from Buenos Aires city. Only C1-f had an evident rodent exposure. The only risk for C1-s was the close contact with C1-f during his prodromal phase. The time between symptom onset was 27 days. Cluster 4 (C4) included a 39-year-old male (C4-a) who shared a 14-hour bus trip with C4-b during the prodromal phase of his illness. C4-c is a work colleague of C4-b and was in contact with C4-b at several times after the latter returned from Neuquén, including visiting him in hospital. The time between onset of symptoms was 15 and 23 days for C4-b and C4-c, respectively. Of the 3 contacts in C4, only C4-a had an evident risk of rodent transmission because he had visited a disease endemic area (Villa La Angostura, southwest of Neuquén City where C4a had holidayed 23 days before onset of symptoms). The nucleotide sequence comparisons in each of the 4 clusters showed 100% identity between cases in the same cluster. ANDV Cent BsAs lineage was characterized from C1, C2 (2/4 cases), and C3, while ANDV Sout was characterized from the 3 C4 cases, although C4-c had never been to the southwestern part of the country. The ANDV Sout lineage has been identified in the southwest region of Argentina. | |
| Martinez 2020 [12] | | Serology - IgM and IgG or Virology – RT-PCR,  Genetic sequencing, Clinical signs and symptoms | | None | H-to-H transmission claimed to be the cause of 33 cases, though possible co-exposure to rodents not measured. Authors claim that only one of the 34 linked cases was caused by exposure to rodents and that the 33 linked cases were all caused by H-to-H transmission. The authors claim that 3 patients were “super-spreaders” in that they each caused 4 or more secondary infections. However, some of the claimed events of H-to-H transmission had minimal contact with a known case (e.g. patient 4). Further, there was no reported investigation of co-exposure to rodents as a possible source. 28 of the 34 cases for which blood samples were available showed 99·8 to 100·0% identity of the ANDV sequences with each other; all but 6 of these lived in the same town (Epuyén). | |
| Wells Arg 1997 [1-4] | | Serology - IgM and IgG or Virology – RT-PCR,  Genetic sequencing, Clinical signs and symptoms | | co-exposure to infected rodents | Sixteen cases (I, A, B, P, L, Q, K, J, H, F, O, N, C, M, E, D), designated Epilink/96, had the same sequence in the M segment G1a, G1b, G2, and in 39 NCR S fragment.[4] The two El Bolsón human case sequences, G and T, belonging to contemporary but non-epidemiologically related patients, differed from Epilink/96. The detection of the same sequence in different people might be explained by infection with a genetically identical hantavirus. The more geographically distant the cases, the more unlikely it is that a unique source of infection or same variant within the rodent local populations occurred. Following this reasoning, infection could have occurred through human-to-human transmission. Of the 16 cases, the authors claim that 2 are likely to be from H-to-H transmission (Doctor D and Case M) due to identical genetic sequence but different geographical area. Doctor D had never been to the area. Case M had never visited El Bolsón but traveled by car for 20 h with a symptomatic infected person (L) and stayed with her infected parents (J and H). No evidence of rodent infestation was found when the car was examined 3 weeks later. No other HPS cases were detected in Buenos Aires or Bariloche during that time. Finding the same sequence in the patients and their contacts in the hospital and the lack of other HPS cases in these cities could suggest human-to-human transmission of the virus.[4] Four cases exposed to cases with HPS in Bariloche hospital (N, O, P, and Q) may be from H-to-H transmission as no other cases of HPS found in Bariloche.[4] However, co-exposure to rodents cannot be ruled out. | |
| Wells 1998 [5] | | Serology - IgM and IgG | | co-exposure to infected rodents | Data do not support H-to-H transmission. Seroprevalence in the random household survey was 1% (3/294). None of these 3 individuals recalled contact with an HPS patient whereas 43/262 IgG negative individuals did report contact with an HPS patient. If HCWs and control-household participants from El Bolsón are included, the community-wide seroprevalence is 0∙6% (three of 470). The authors conclude that "no evidence for asymptomatic infection among HCWs was found." | |
| Chile – Andes virus | | | |  |  | |
| Castillo 2004 [13, 14] | | Serology – IgG | | PPE, co-exposure to infected rodents | No evidence for H-to-H transmission. The prevalence of antibodies against hantavirus was 1∙9% (95% CI = 0∙34−6∙3%) in family member contacts compared with 0∙0% (95% CI = 0−3∙2%) in the HCWs. The seroprevalence among the family members was similar to the seroprevalence of 2∙5−7∙5% in the rural communities where they live. Most family contacts had potential environmental exposure to rodents in their homes. | |
| Martinez 2014 [15] | | Serology - IgM and IgG, Virology – RT-PCR,  Genetic sequencing | | co-exposure to infected rodents | H-to-H transmission appeared likely in 4 of the 5 cases in the cluster. Case-patient A (index case) lived in a small settlement near Corral. His main risk activity was the cleaning of a home cellar where he was moving tiles on February 5. B (nurse at hospital 1) developed symptoms 19-21 days after caring for A and 25-26 days after possible environmental exposure (camping site). C (wife of A) developed symptoms 22-25 days after exposure to A and 41 days after environmental exposure (cellar). D (housekeeper at hospital 1) developed symptoms 18-20 days after caring for A (helped B change his clothes, sheets, and bedclothes for washing) and 7-45 days after possible environmental exposure (lived in a rural area). E (husband of B) developed symptoms 13-27 days after exposure to B and 41-42 days after possible environmental exposure (camping). Genetic sequencing showed 99-100% identity of the 5 patients in the cluster, though all lived in the same geographic area. Rodent trapping around cellar and camping site conducted 2-3 months after diagnosis of A were negative. The authors claim that the evidence: "strongly supports the conclusion that 4 of 5 cases resulted from person-to-person transmission of ANDV, including 2 cases of nosocomial transmission." | |
| Toro 1998 [16] | | Serology - IgM and IgG,  Immunohistochemistry, Virology – RT-PCR,  Genetic sequencing (in some) | | co-exposure to infected rodents | H-to-H transmission appeared likely for Cluster 3, possible for Cluster 1 (co-exposure to rodents cannot be ruled out). Genetic sequencing of viral RNA from cases of immediate family members from Clusters 1 and 2 demonstrated 3∙6% divergence in a 167-nucleotide G2 fragment between the clusters. However, the genetic sequence was identical within each of the two clusters, except for the brother-in-law in Cluster 1. No analysis was presented for Cluster 3. Cluster 1 - the immediate family members became ill 12, 19, and 28 days after leaving the family homestead, and the intervals between the onset of the index and later cases were 18, 25, and 34 days. They returned home to collect belongings 5 days after leaving. Evidence for H-to-H transmission is based on time between infections for each of these family members. The brother-in-law continued to reside intermittently in the house. Cluster 2 - included all four family members of a household, who became ill within 5 days of each other. Cluster 3 - a husband, who worked in a rural area, became ill 12 days after returning to his family home. His wife, who remained in the family home in urban Coyhaique, became ill 22 days after the onset of the husband's symptoms. She had not traveled outside the town of Coyhaique during the previous 12 months and reported no exposure to rodents or their excreta. Among 53 contacts of 14 patients, 2 (3∙8%) had serologic evidence of an acute infection (one had no illness, and another [described previously in cluster 2] had a mild febrile illness without pulmonary disease that did not meet the HPS case definition), and one was IgG positive. | |
| Germany - PUUV and DOBV | | | |  | |  |
| Hofmann 2012 [17] | | Serology – IgM and IgG, Virology – RT-PCR | | None | No evidence for H-to-H (vertical) transmission. After delivery, no hantavirus RNA was found by RT-PCR in the cord blood samples from all four infants. The IgG positivities of the mothers were confirmed for the 3 babies delivered at term (cord blood), however, there were no corresponding IgM titers. This indicated the occurrence of transplacental IgG transfer in the absence of de-novo IgM synthesis in the fetus. In the cord blood serum from the preterm newborn delivered by caesarian section during gestational week 28, neither hantaviral IgM nor IgG was detected. At 8-12 months of age no hantavirus IgG, IgM, or RNA was detected in the serum of 3 infants initially IgG-positive. Moreover, no symptoms of hantavirus disease or other abnormalities were found in any of the babies. | |
|  |  | |  |  |  |  |
| South Korea – Hantaan virus | | | |  | |  |
| Park 2019 [18] | | Serology – IgG | | PPE | No evidence for H-to-H transmission. Two of the HCWs (a doctor and nurse) who cared for the patient on the general ward and who participated in cardiopulmonary resuscitation 3 days after admission had symptoms corresponding to a febrile illness 21-23 days after exposure and increased IgG titers 28 and 30 days after exposure. Neither had used PPE. The other 4 HCWs (a doctor and 3 nurses) did not have increased IgG titers but their contact with the patient was during the renal phase and all used some form of PPE. The authors conclude: "we found no evidence of Hantaan virus person-to-person transmission in HCWs exposed to the index case." | |
| United States of America - Sin Nombre virus | | | |  | | |
| Howard 1999 [19] | | Virology – RT-PCR, Immunohistochemistry | | None | No evidence for H-to-H (vertical) transmission. 2 babies died in utero and 1 was born prematurely and died after discharge from hospital. Gross, microscopic, and immunohistochemical examination for hantavirus antigen were done on 2 fetal autopsies and 3 placentas showing no evidence of transplacental hantavirus transmission. There was no serological evidence of conversion in the 3 surviving children (only 2 measured post-discharge). None of the women had evidence of abnormal fetal development, distress, or complications of their pregnancies before presentation. | |
| Wells USA 1997 [20] | | Serology - IgM and IgG, or Immunohistochemistry | | co-exposure to infected rodents | No evidence for H-to-H transmission. The interval between cases was much shorter than 8 weeks in each instance, and contact between the linked patients from clusters 1, 3, and 4 occurred before patients were admitted to a hospital. All infected persons could have been exposed to rodent excreta in their home or work environments. Exposure risk was documented by results of rodent trapping in clusters 2, 3, and 4. In clusters 1 and 5, the men had been sleeping and working in rural environments where rodent infestation was noted, but trapping was not performed. Based on the U.S. hantavirus case registry, of the 160 confirmed HPS patients until the publication of this study household and social contacts (n=320) of 40 of these patients have been tested: 310/320 (96∙9%) had no serologic evidence of hantavirus infection; 3 had IgG that reacted to SNV antigen (SNV-IgG) but no illness, 1 had SNV-IgG and was diagnosed with HPS retrospectively, who was part of Cluster 1; 6 had SNV-IgM: 4 had confirmed cases of HPS, and correspond to Clusters 2 & 5, and the other 2 had a clinical course not consistent with that of HPS (Clusters 3 & 4). | |

HCW – healthcare workers; HPS – hantavirus pulmonary syndrome; H-to-H – human-to-human; PPE – personal protective equipment.

^a^ If a study had more than one reference, we awarded one reference the status of primary reference. The Study ID was taken from the first author of the primary reference and year of publication.

# **References**

1. Wells RM, Sosa Estani S, Yadon ZE, et al. An unusual hantavirus outbreak in southern Argentina: person-to-person transmission? Hantavirus Pulmonary Syndrome Study Group for Patagonia. Emerg Infect Dis **1997**; 3:171-4.

2. Cantoni G, Lazaro M, Resa A, et al. Hantavirus pulmonary syndrome in the Province of Rio Negro, Argentina, 1993-1996. Rev Inst Med Trop Sao Paulo **1997**; 39:191-6.

3. Enria D, Padula P, Segura EL, et al. Hantavirus pulmonary syndrome in Argentina. Possibility of person to person transmission. Medicina (B Aires) **1996**; 56:709-11.

4. Padula PJ, Edelstein A, Miguel SD, Lopez NM, Rossi CM, Rabinovich RD. Hantavirus pulmonary syndrome outbreak in Argentina: molecular evidence for person-to-person transmission of Andes virus. Virology **1998**; 241:323-30.

5. Wells RM, Sosa Estani S, Yadon ZE, et al. Seroprevalence of antibodies to hantavirus in health care workers and other residents of southern Argentina. Clin Infect Dis **1998**; 27:895-6.

6. Alonso DO, Perez-Sautu U, Bellomo CM, et al. Person-to-person transmission of Andes virus in hantavirus pulmonary syndrome, Argentina, 2014. Emerg Infect Dis **2020**; 26:756-9.

7. Iglesias AA, Bellomo CM, Martinez VP. [Hantavirus pulmonary syndrome in Buenos Aires, 2009-2014]. Medicina (B Aires) **2016**; 76:1-9.

8. Lazaro ME, Cantoni GE, Calanni LM, et al. Clusters of hantavirus infection, southern Argentina. Emerg Infect Dis **2007**; 13:104-10.

9. Martinez VP, Bellomo C, San Juan J, et al. Person-to-person transmission of Andes virus. Emerg Infect Dis **2005**; 11:1848-53.

10. Martinez VP, Bellomo CM, Cacace ML, Suarez P, Bogni L, Padula PJ. Hantavirus pulmonary syndrome in Argentina, 1995-2008. Emerg Infect Dis **2010**; 16:1853-60.

11. Pinna DM, Martinez VP, Bellomo CM, Lopez C, Padula P. [New epidemiologic and molecular evidence of person to person transmission of hantavirus Andes Sout]. Medicina (B Aires) **2004**; 64:43-6.

12. Martínez VP, Di Paola N, Alonso DO, et al. “Super-Spreaders” and Person-to-Person Transmission of Andes Virus in Argentina. N Engl J Med **2020**; 383:2230-41.

13. Castillo C, Mardones J, Villagra E. [Prevalence of anti-hantavirus antibodies in health care personnel in direct contact with patients with hantavirus pulmonary syndrome in Temuco, Chile 1997 to 1999]. Rev Med Chil **2000**; 128:735-9.

14. Castillo C, Villagra E, Sanhueza L, Ferres M, Mardones J, Mertz GJ. Prevalence of antibodies to hantavirus among family and health care worker contacts of persons with hantavirus cardiopulmonary syndrome: lack of evidence for nosocomial transmission of Andes virus to health care workers in Chile. Am J Trop Med Hyg **2004**; 70:302-4.

15. Martinez-Valdebenito C, Calvo M, Vial C, et al. Person-to-person household and nosocomial transmission of andes hantavirus, Southern Chile, 2011. Emerg Infect Dis **2014**; 20:1629-36.

16. Toro J, Vega JD, Khan AS, et al. An outbreak of hantavirus pulmonary syndrome, Chile, 1997. Emerg Infect Dis **1998**; 4:687-94.

17. Hofmann J, Fuhrer A, Bolz M, et al. Hantavirus infections by Puumala or Dobrava-Belgrade virus in pregnant women. J Clin Virol **2012**; 55:266-9.

18. Park JW, Joo EJ, Cheong HS. Possibility of nosocomial person-to-person transmission of hemorrhagic fever with renal syndrome. Infect Control Hosp Epidemiol **2019**; 40:1329-31.

19. Howard MJ, Doyle TJ, Koster FT, et al. Hantavirus pulmonary syndrome in pregnancy. Clin Infect Dis **1999**; 29:1538-44.

20. Wells RM, Young J, Williams RJ, et al. Hantavirus transmission in the United States. Emerg Infect Dis **1997**; 3:361-5.
